# Supplementary material for: High-parametric protein maps reveal the spatial organization in early-developing human lung
Source: Nat Commun. 2024 Oct 30;15:9381. doi: 10.1038/s41467-024-53752-x (PMC11525936; doi:10.1038/s41467-024-53752-x)
Supplement: Supplementary file 1 — Supplementary Information [file 41467_2024_53752_MOESM1_ESM.pdf]

SUPPLEMENTARY INFORMATION

High-parametric protein maps reveal the spatial organization in early-developing human lung

Sanem Sariyar[1,2], Alexandros Sountoulidis[1,3], Jan Niklas Hansen[1,2,4], Sergio Marco Salas[1,5], Mariya Mardamshina[1,2], Anna Martinez Casals[1,2,4], Frederic Ballllosera Navarro[1,2,4], Zaneta Andrusivova[1,6], Xiaofei Li[7], Paulo Czarnewski[1,6], Joakim Lundeberg[1,6], Sten Linnarsson[8], Mats Nilsson[1,5], Erik Sundström[7], Christos Samakovlis[1,3,9], Emma Lundberg[1,2,4,10,*,#], Burcu Ayoglu[1,2,*,#]

[1]Science for Life Laboratory, Solna, Sweden
[2]Department of Protein Science, KTH - Royal Institute of Technology, Stockholm, Sweden
[3]Department of Molecular Biosciences, Wenner-Gren Institute, Stockholm University, Stockholm, Sweden
[4]Department of Bioengineering, Stanford University, Stanford, CA, USA
[5]Department of Biochemistry and Biophysics, Stockholm University, Stockholm, Sweden.
[6]Department of Gene Technology, KTH - Royal Institute of Technology, Stockholm, Sweden
[7]Department of Neurobiology, Care Sciences and Society, Karolinska Institutet, Stockholm, Sweden
[8]Department of Medical Biochemistry and Biophysics, Karolinska Institute, Stockholm, Sweden
[9]Molecular Pneumology, Cardiopulmonary Institute, Justus Liebig University, Giessen, Germany
[10]Department of Pathology, Stanford University, Stanford, CA, USA

* Equal contribution
\# Correspondence should be addressed to: burcu.ayoglu@scilifelab.se or emmalu@stanford.edu

Supplementary Discussion Point

A number of cell type clusters in analyses shown in ***Figure 2A-B*** and ***Figure 3A-B***, ***Figure 4***, and all related supplementary figures were not represented uniformly across all the weeks due to a number of technical reasons:

As shown by the podoplanin (PDPN) antibody staining patterns in ***Figure 1D,*** mesothelial cells were indeed present at all five developmental weeks. However, as detailed in ***Supplementary Figure 4A***, in the relatively larger tissues of the 8.5 week and all later-stage lung samples, we experienced uneven signal intensity for various markers in peripheral regions, causing issues in downstream clustering after image segmentation. Thus, we removed these peripheral regions before downstream data analysis. This impacted the representation of mesothelial cells in all weeks except the week 6 in the downstream analyses.

Chondroblasts were missing in the 12 week old sample because the section presented for the 12 week old sample originated from a relatively more peripheral region of the organ, representing more distal parts of the airway network as compared to other samples.

For the pericytes and the airway fibroblasts, we did not have single specific markers for these cell types. Instead, similar to neuronal cells, we used a combination of several markers that provided clusters with characteristic signatures aligning with the positioning and morphology of these cells in the tissue images. This approach, while demonstrating the strength of multi-parametric image analysis at the protein level, is prone to underperform if one or more of the markers do not perform equally well (*e.g.* ACTA2 in week 12 compared to weeks 11 and 13), affecting the clustering performance.

Adventitial fibroblasts are localized within the bronchovascular bundles of the lung[1]. Among the analyzed sections, only those from the week 11 sample contained such a central part of the organ. This provides an explanation about their absence in more peripheral lung sections.

Supplementary Table 1. List of and detailed information on all evaluated antibodies.
Detailed information such as catalogue number, clone ID, target, selection reason, conjugated barcode, indirect IF dilutions and tested tissues, failed steps for all evaluated antibodies in the study. In the “Failed step” column, the step where antibodies failed is reported according to the evaluation after the indirect IF, conjugation or multiplexed imaging run.

LIST OF ALL TESTED ANTIBODIES

Antibodies	Catalog number	Clone	RRID	Target	Selection Reason	Barcodes of Akoya Immune Panel Antibodies	Regular IF Dilution	Regular IF Tested Tissue	In-House Conjugated/Barcode	Failed Step	Phenocycler Dilutions	Worked in the run
ACTA2	ab119952	4A4	AB_10901360	Smooth muscle actin	sc-RNAseq	N/A	1/200	Fetal lung 13 pcw	BX028	N/A	1/70	Yes
ASCL1	ab240385	EPR19840		Neuroendocrine cells	sc-RNAseq	N/A	1/50- 1/100	Fetal Lung 8.5 pcw	N/A	Failed in Regular IF	N/A	N/A
CD104	4250008	58XB4		Epithelial cells	Akoya Immune Panel	BX005	N/A	N/A	N/A	Failed in the run	N/A	N/A
CD11c	4350012	S-HCL-3		Dendritic cells/ macrophages	Akoya Immune Panel	BX027	N/A	N/A	N/A	Failed in the run	N/A	N/A
CD123	# 14-1239-82	6H6	AB_467453	Endothelial cells&Hematopoetic stem cells	Literature/sc-RNAseq	N/A	1/100	Tonsil	BX54	N/A	1/100	Yes
CD14	# 14-0149-82	61D3		Monocyte	Literature/sc-RNAseq	N/A	1/50	Tonsil/Spleen	BX006	Failed in the run	N/A	N/A
CD144	# 14-1449-82	16B1	AB_467495	Endothelial cells&Hematopoetic stem cells	Literature/sc-RNAseq	N/A	1/100	Pancreas	BX016	N/A	1/100	Yes
CD163	HPA046404			Macrophages	Literature/sc-RNAseq	N/A	1/130	Fetal lung 13 pcw	BX025	Failed in Conjugation	N/A	N/A
CD163	# MA5-17716	GHI/61	AB_2539106	Macrophages	Literature/sc-RNAseq	N/A	1/50	Fetal lung 13 pcw	BX049/ BX005	N/A	1/50	Yes
CD19	4350003	HIB19		B cells	Akoya Immune Panel	BX003	N/A	N/A	N/A	N/A	1/100	Yes
CD2	4250005	RPA-2.10		T cells	Akoya Immune Panel	BX002	N/A	N/A	N/A	Failed in the run	N/A	N/A
CD20	# 14-0202-82	L26		B cells	Literature/sc-RNAseq	N/A	1/100- 1/1000	Spleen	N/A	Failed in Regular IF	N/A	N/A
CD21	4150009	Bu32		B cells	Akoya Immune Panel	BX013	N/A	N/A	N/A	Failed in the run	N/A	N/A
CD278	4250013	C398.4A		Activated helper and cytotoxic cells	Akoya Immune Panel	BX017	N/A	N/A	N/A	Failed in the run	N/A	N/A
CD279	4250010	EH12.2H7		T cells	Akoya Immune Panel	BX014	N/A	N/A	N/A	Failed in the run	N/A	N/A
CD3	4350008	UCHT1		T cells	Akoya Immune Panel	BX015	N/A	N/A	N/A	N/A	1/200	Yes
CD31	4250009	WM59		Endothelial cells	Akoya Immune Panel	BX032	N/A	N/A	N/A	N/A	1/200	Yes
CD34	4250020	561		Endothelial cells	Akoya Immune Panel	BX035	N/A	N/A	N/A	N/A	1/200	Yes
CD38	4150007	FF		General immune cells marker	Akoya Immune Panel	BX007	N/A	N/A	N/A	Failed in the run	N/A	N/A
CD4	4350010	SK3		Helper T cells	Akoya Immune Panel	BX021	N/A	N/A	N/A	N/A	1/200	Yes
CD41	HPA031169			Megakaryocytes	Literature/sc-RNAseq	N/A	1/250	Fetal lung 13 pcw	BX041	Failed in Conjugation	N/A	N/A
CD44	# MA4400	Hermes-1	AB_223517	Hematopoetic stem cells	Literature/sc-RNAseq	N/A	1/50	Fetal Lung 12 pcw	BX020	N/A	1/100	Yes
CD45	4150003	HI30		General immune cells marker	Akoya Immune Panel	BX001	N/A	N/A	N/A	N/A	1/100	Yes
CD56	# MA1-06801	123C3		NK cells	Literature/sc-RNAseq	N/A	1/100	Pancreas	BX029	Regular IF	N/A	N/A
CD56	ab251595	CAL53		NK cells	Literature/sc-RNAseq	N/A	1/50	Fetal lung 13 pcw	BX029	N/A	1/50	Yes
CD59	# MA5-17046	8D2B8		Lineage marker	Literature	N/A	1/200	Pancreas	BX033	Failed in the run	N/A	N/A
CD68	# 14-0688-82	KP1	AB_11151139	Macrophage	Literature/sc-RNAseq	N/A	1/100	Tonsil	BX10	N/A	1/100	Yes
CD69	4250022	FN50		Lymphocyte activation marker	Akoya Immune Panel	BX041	N/A	N/A	N/A	Failed in the run	N/A	N/A
CD7	4150022	CD7-6B7		Differentiation marker for T cells	Akoya Immune Panel	BX025	N/A	N/A	N/A	Failed in the run	N/A	N/A
CD8	4150004	SK1		Cytotoxic T cells	Akoya Immune Panel	BX004	N/A	N/A	N/A	Failed in the run	N/A	N/A
CD9	4150016	HI9a		Myeloid cell lineage cells	Akoya Immune Panel	BX028	N/A	N/A	N/A	Failed in the run	N/A	N/A
CD90	4150021	5E10		Stem cells	Akoya Immune Panel	BX022	N/A	N/A	N/A	N/A	1/200	Yes
CKIT	#348800	2B8		Hematopoetic stem cells	Literature/sc-RNAseq	N/A	1/125	Mouse cerebellum	BX45	Failed in the run	N/A	N/A
CKIT	HPA073252			Hematopoetic stem cells	Literature/sc-RNAseq	N/A	1/30	Fetal lung 13 pcw	BX050	Failed in Conjugation	N/A	N/A
CLDN5	# 35-2500	4C3C2	AB_2533200	Endothelial cells	sc-RNAseq	N/A	1/100	Fetal lung 7 w	BX041	N/A	1/100	Yes
COL1A1	MAB6220-100	# 816161		Mesenchymal	sc-RNAseq	N/A	1/100	Fetal lung 13 pcw	BX002	N/A	1/300	Yes
CSF1R	ab240265	SP211		Macrophages	sc-RNAseq	N/A	1/100	Fetal lung 13 pcw	BX017/BX036	Failed in the run	N/A	N/A
CXCR4	# 35-8800	12G5		Hematopoetic stem cells	Literature/sc-RNAseq	N/A	1/50-1/500	Spleen	N/A	Failed in Regular IF	N/A	N/A
DCN	HPA003315			Mesenchymal	sc-RNAseq	N/A	1/200	Fetal lung 13 pcw	BX013	N/A	1/50	Yes
Ecadherin	4250021	4A2C7		Epithelial Cells	Akoya FFPE Panel	BX014	N/A	N/A	N/A	N/A	1/200	Yes
EPCAM	#14932682	1B7	AB_795876	Epithelial cells	sc-RNAseq	N/A	1/100	Fetal lung 13 pcw	BX042	N/A	1/400	Yes
FLT3	# PA5-34448			Hematopoetic stem cells	Literature/sc-RNAseq	N/A	1/50-1/500	Spleen	N/A	Failed in Regular IF	N/A	N/A
GP1BA	HPA013316			Megakaryocytes	Literature/sc-RNAseq	N/A	1/110	Fetal lung 13 pcw	BX045	Failed in Conjugation	N/A	N/A
GPC3	# MA5-17083	9C2		Alveolar fibroblast	sc-RNAseq	N/A	1/100	Fetal lung 7 w	N/A	Failed in Regular IF	N/A	N/A
GRHL2	# MA5-31388	CL3760		Mesenchyme	sc-RNAseq	N/A	1/250	Fetal lung 13 pcw	BX024	Failed in Regular IF	N/A	N/A
HHIP	H00064399-M01	5D11		Lung airways	sc-RNAseq	N/A	1/50	Fetal lung 13 pcw	N/A	Failed in Regular IF	N/A	N/A
HLA-DR	4250006	L243		Antigen presenting cells	Akoya Immune Panel	BX026	N/A	N/A	N/A	N/A	1/200	Yes
HPGD	NB200-179			Aerocytes	sc-RNAseq	N/A	1/100	Fetal lung 7 w	N/A	Failed in Regular IF	N/A	N/A
IBA1	ab221790	EPR16589		Macrophages	sc-RNAseq	N/A	1/50	Fetal Lung 12 pcw	BX30, BX027	Failed in Conjugation	N/A	N/A
IBA1	ab220815	EPR16588		Macrophages	sc-RNAseq	N/A	1/100	Fetal lung 7 w	BX036	Failed in the run	N/A	N/A
Ki67	4250019	B56		Proliferating cells	Akoya Immune Panel	BX047	N/A	N/A	N/A	N/A	1/200	Yes
LYZ	ab185129	EPR2994(2)		Macrophages	sc-RNAseq	N/A	1/1000	Fetal lung 7 w	BX050	Failed in Conjugation	N/A	N/A
MCM3	SAB1404055	3E1		Proliferating cells	sc-RNAseq	N/A	1/50	Fetal lung 13 pcw	N/A	Failed in Regular IF	N/A	N/A
MRC1	ab64693	N/A	AB_1523910	Macrophages	sc-RNAseq	N/A	1/500	Fetal lung 13 pcw	BX030	N/A	1/100	Yes
NRXN1	#703650	22H29L23		Cell adhesion	sc-RNAseq	N/A	1/100	Fetal lung 13 pcw	N/A	Failed in Regular IF	N/A	N/A
Pan-cytokeratin	4150020	AE-1/AE-3		Epithelial Cells	Akoya Immune Panel	BX019	N/A	N/A	N/A	N/A	1/300	Yes
PDGFRB	MA535288	ARC0009		Mesenchymal	sc-RNAseq	N/A	1/100	Fetal lung 13 pcw	N/A	Failed in Regular IF	N/A	N/A
PDGFRB	AF385			Mesenchymal	sc-RNAseq	N/A	1/100	Fetal lung 13 pcw	N/A	Failed in Regular IF	N/A	N/A
Podoplanin	4250004	NC-08		Lymphatic endothelial cells	Akoya Immune Panel	BX023	N/A	N/A	N/A	N/A	1/200	Yes
PRX	ab278083	EPR24150-36		Capillaries	sc-RNAseq	N/A	1/100	Fetal lung 7 w	BX052	N/A	1/100	Yes
PSAP	ab249391	EPR10784(B)		Macrophages	sc-RNAseq	N/A	1/50	Fetal lung 13 pcw	BX055	Unsure Regular IF & Failed conjugation	N/A	N/A
RUNX1	SAB1412471	3A1		Hematopoetic stem cells	Literature/sc-RNAseq	N/A	1/50	Fetal lung 13 pcw, Spleen	N/A	Failed in Regular IF	N/A	N/A
RUNX1	H00000861-M06	M06		Hematopoetic stem cells	Literature/sc-RNAseq	N/A	1/300	Fetal Lung 12 pcw	N/A	Failed in Regular IF	N/A	N/A
RUNX1	HPA004176			Hematopoetic stem cells	Literature/sc-RNAseq	N/A	1/25	Fetal lung 7 pcw	N/A	Failed in Regular IF	N/A	N/A
Serglycin	# PA5-50794			Hematopoetic stem cells	Literature/sc-RNAseq	N/A	1/100	Fetal lung 13 pcw	BX025- BX030	Failed in Conjugation	N/A	N/A
SFTPC	H00006440-M01	4A10		Type 2 alveolar cells	sc-RNAseq	N/A	1/100	Fetal lung 13 pcw	N/A	Failed in Regular IF	N/A	N/A
SOX2	14-9811-82	Btjce	AB_11219471	Proximal epithelial cells	sc-RNAseq	N/A	1/250	Fetal Lung 8.5 pcw	BX024	N/A	1/200	Yes
SOX9	MA5-17177	1B11		Distal epithelial cells	sc-RNAseq	N/A	1/200	Fetal Lung 8.5 pcw	N/A	Failed in Regular IF	N/A	N/A
SOX9	AF3075		AB_2194160	Distal epithelial cells	sc-RNAseq	N/A	1/500	Fetal lung 7 w	BX033	N/A	1/250	Yes
TTF1	ab216648	EP1584Y		Airway cells	sc-RNAseq	N/A	1/250	Whole embryo	BX007	N/A	1/100	Yes
Vimentin	677802	O91D3	AB_2565982	Mesenchymal	sc-RNAseq	N/A	1/50	Fetal lung 13 pcw	BX045	N/A	1/300	Yes
WT1	# MA1-46028	6F-H2	AB_962464	Mesothelium	sc-RNAseq	N/A	1/400	Fetal lung 13 pcw	BX006	N/A	1/50	Yes

Supplementary Table 2. Values for background subtraction. Values for background subtraction for all the markers and developmental weeks.

	6 pcw	8.5 pcw	11 pcw	12 pcw	13 pcw
**ACTA2**	0.4576	13.494	0.8668	0.06	0.4301
**CD3**	3.3022	0.385	8.1543	3.2307	1.3552
**CD4**	23.0351	2.378	0.5302	6.6352	3.1262
**CD19**	1.9756	15.017	0.0528	1.8601	3.2164
**CD31**	0.1232	0.791	1.4586	5.507	8.179
**CD34**	0.0418	0.152	0.2816	0.461	3.198
**CD44**	3.3792	4.9	4.908	4.4517	24.0
**CD45**	1.5235	1.873	4.2471	3.4507	0.0737
**CD56**	0.1518	2.386	2.2077	0.0517	0.5819
**CD68**	20.372	14.277	20.3258	17.8244	9.886
**CD90**	10.0507	7.938	7.287	15.1866	7.0
**CD123**	0.8	0.230	2.3969	1.6126	0.1628
**CD144**	4.8	4.719	3.5992	6.6	1.782
**CD163**	2.1252	0.773	0.3883	5.6958	4.5078
**CLDN5**	6.3481	11.708	0.3179	0.2343	0.6094
**COL1A1**	3.105	3.083	0.8679	1.3849	1.0912
**DCN**	6.0	10.0	10.0	6.5	8.9
**Ecadherin**	0.3223	0.068	0.2211	0.9812	0.5115
**EPCAM**	1.9305	2.750	7.5438	6.864	10.9747
**HLADR**	0.3509	0.114	1.2001	5.124	1.3376
**KI67**	8.0	1.4	0.1298	0.0671	0.143
**MRC1**	4.8939	0.286	0.1221	15.609	2.2374
**Pancytokeratin**	0.7854	2.192	3.0316	0.2992	0.022
**Podoplanin**	0.8602	11.080	3.6212	1.5257	3.4892
**PRX**	0.055	0.025	0.143	0.0209	0.0209
**SOX2**	1.0604	0.232	0.4125	0.0363	3.0932
**SOX9**	7.9354	16.633	0.5709	4.0546	1.2672
**TTF1**	1.8656	5.008	6.1809	1.9426	0.198
**Vimentin**	8.9419	8.020	0.9834	4.4363	3.9633
**WT1**	13.0	9.0	3.5	8.437	0.4741

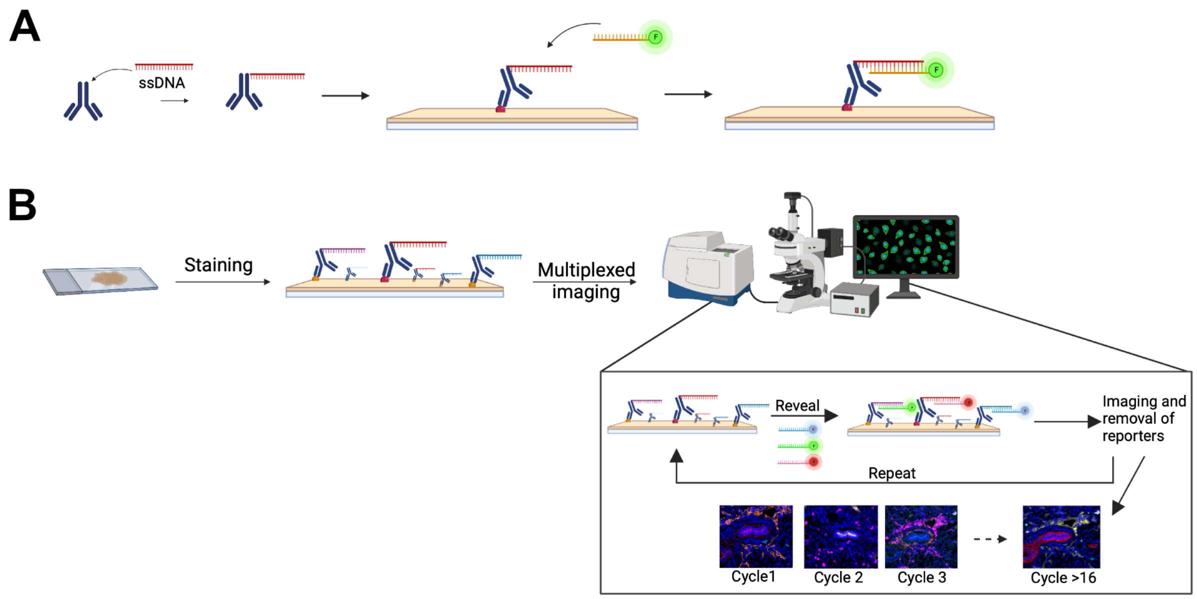

Supplementary Figure 1. High-parametric tissue imaging workflow. A) Steps of antibody conjugation and conjugation validation. Antibodies were conjugated with single-stranded DNA (ssDNA) and validation of the conjugated antibodies was done with reporters (complementary DNA strands conjugated to fluorophores). **B)** Staining the tissue with conjugated antibodies. During each cycle of the automated image acquisition run, up to three reporters were revealed to visualize up to three different antibodies. After each imaging cycle, the reporters were removed to reveal the next set of reporters. (Created in BioRender. Ayoglu, B. (2022) BioRender.com/l411753)

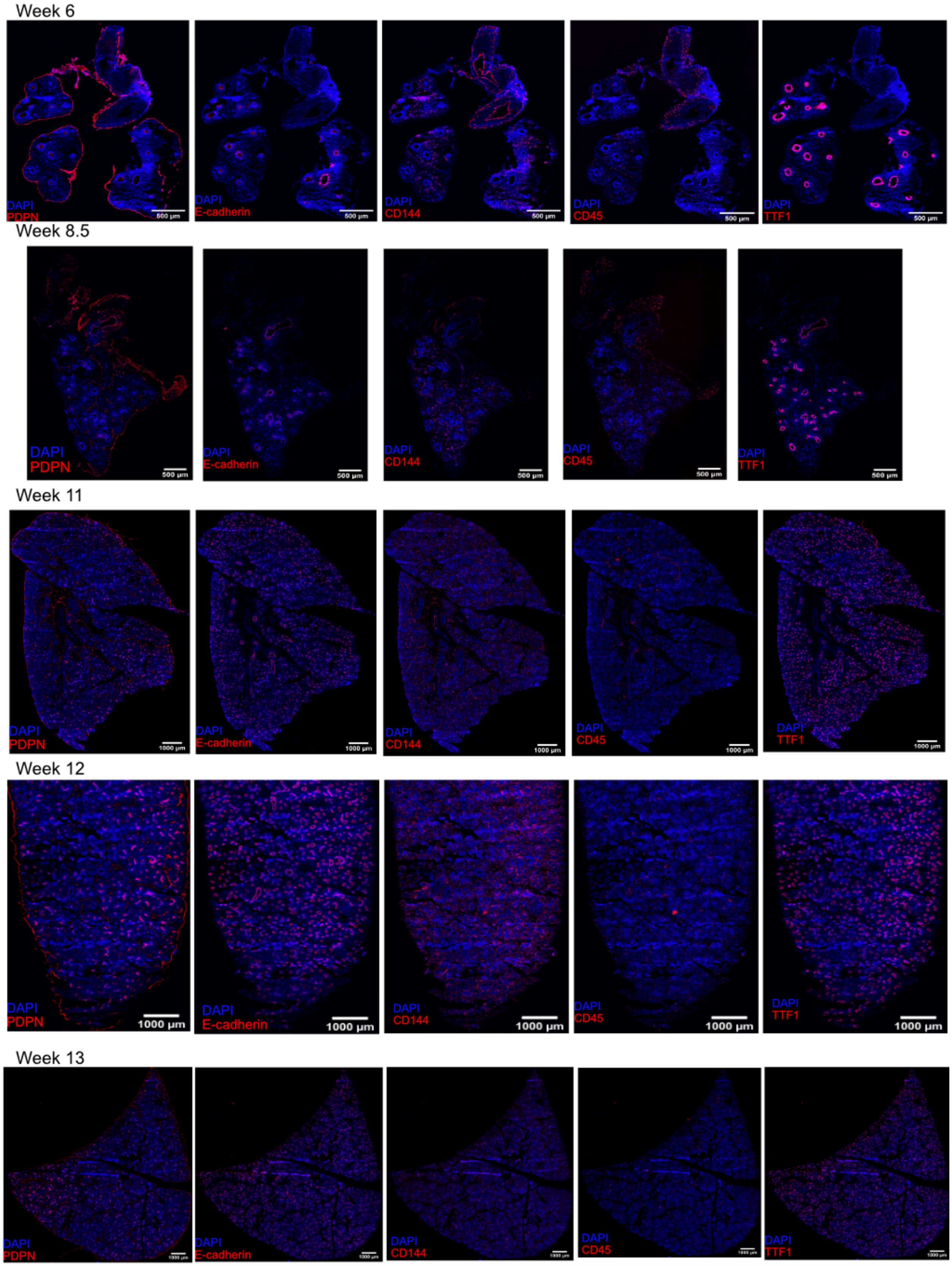
Week 6
DAPI
PDPN
500 µm
DAPI
E-cadherin
500 µm
DAPI
CD144
500 µm
DAPI
CD45
500 µm
DAPI
TTF1
500 µm
Week 8.5
DAPI
PDPN
500 µm
DAPI
E-cadherin
500 µm
DAPI
CD144
500 µm
DAPI
CD45
500 µm
DAPI
TTF1
500 µm
Week 11
DAPI
PDPN
1000 µm
DAPI
E-cadherin
1000 µm
DAPI
CD144
1000 µm
DAPI
CD45
1000 µm
DAPI
TTF1
1000 µm
Week 12
DAPI
PDPN
1000 µm
DAPI
E-cadherin
1000 µm
DAPI
CD144
1000 µm
DAPI
CD45
1000 µm
DAPI
TTF1
1000 µm
Week 13
DAPI
PDPN
DAPI
E-cadherin
DAPI
CD144
DAPI
CD45
DAPI
TTF1

Supplementary Figure 2. Original imaging area for the lung tissues from different developmental weeks. Staining patterns of PDPN (lymphatic endothelial and mesothelial marker), CDH1 (E-Cadherin, epithelial marker), CD144 (endothelial marker), CD45 (general immune marker) and TTF1 (epithelial marker) are shown in red on the original imaging area. Images represent a single donor for each developmental week. Scale bar corresponds to 500 μm in weeks 6 and 8.5, and to 1000 μm in weeks 11, 12 and 13.

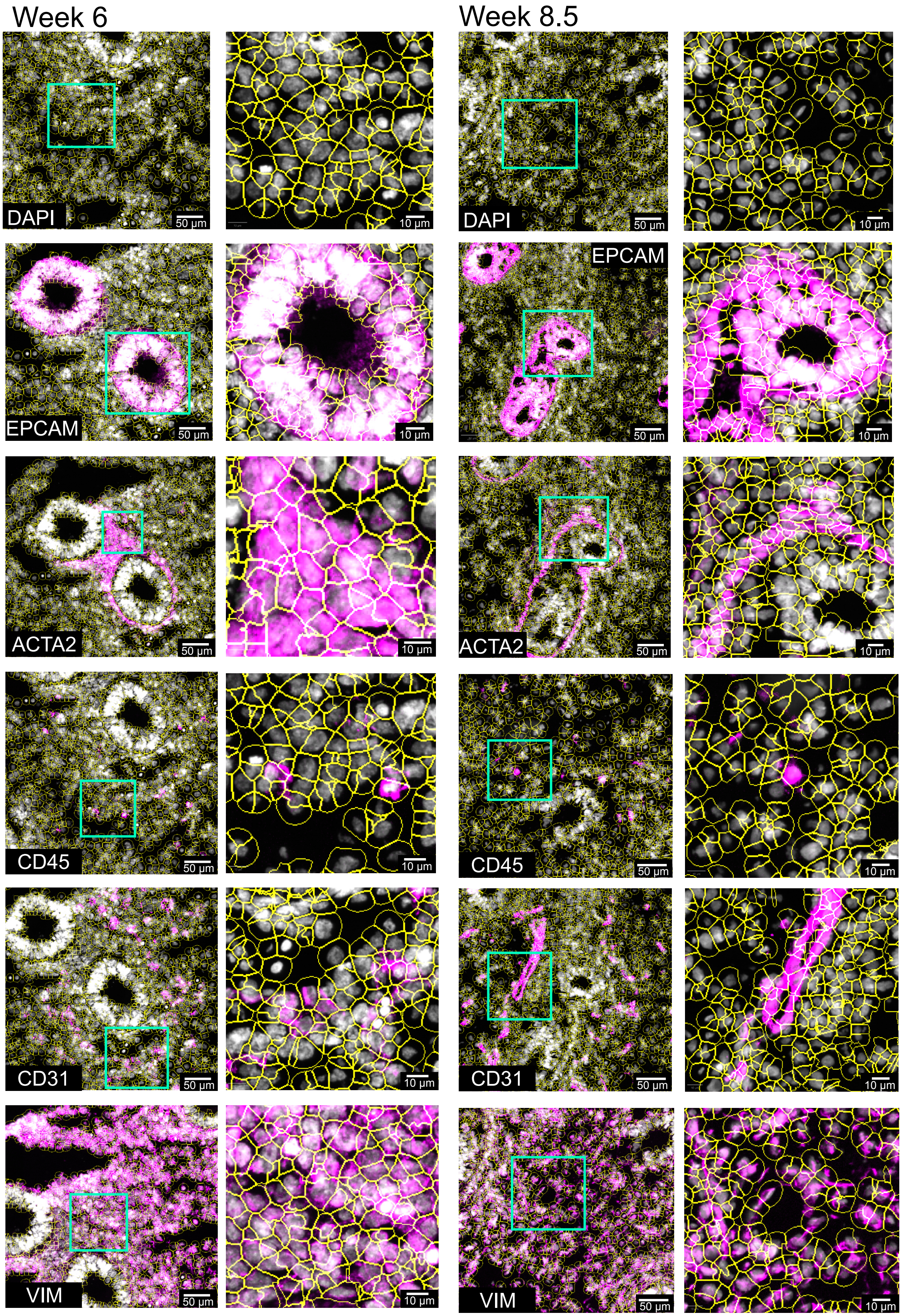
Week 6
Week 8.5
DAPI
EPCAM
ACTA2
CD45
CD31
VIM
50 µm
10 µm

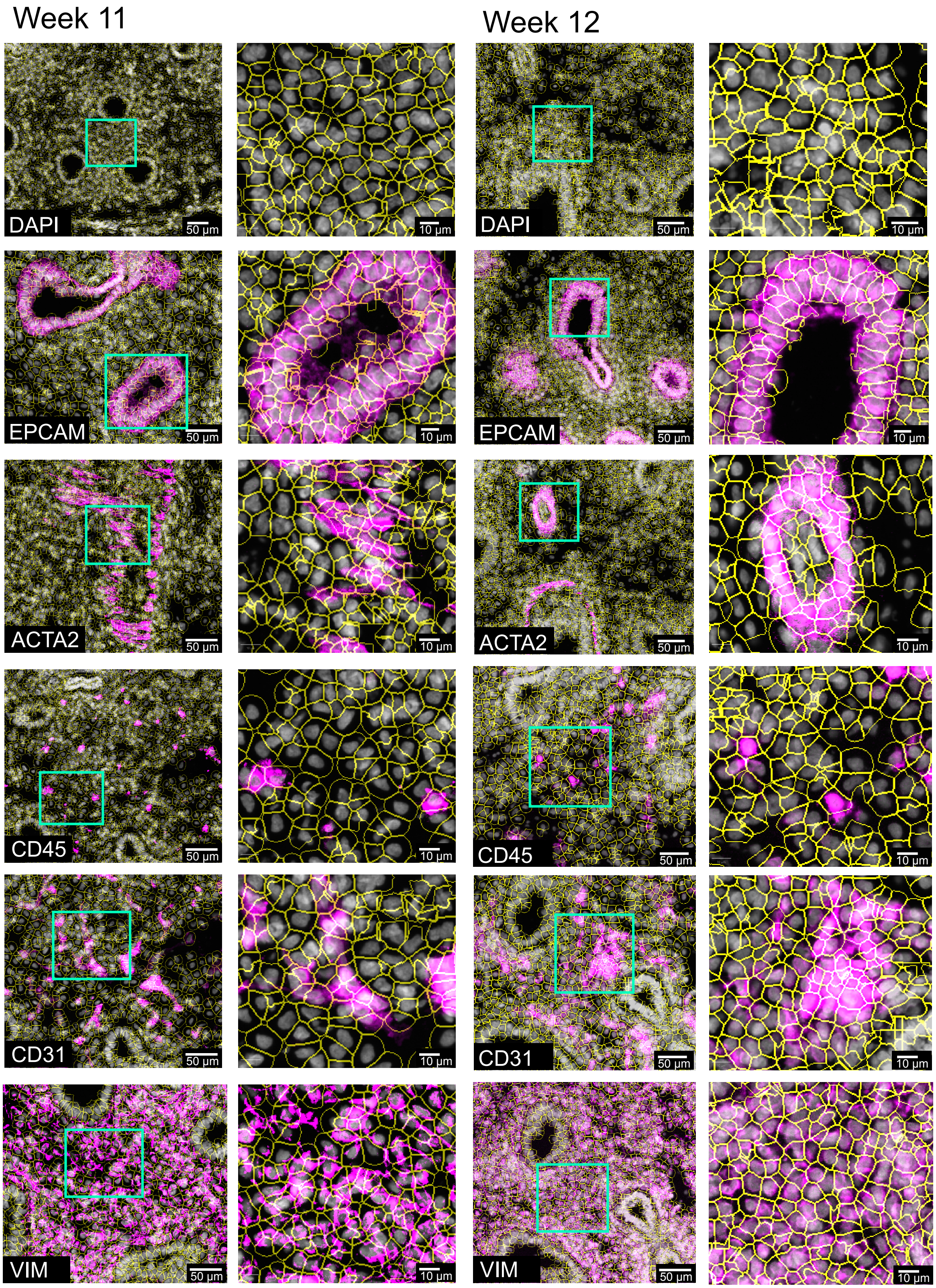

Week 11
Week 12
DAPI
50 µm
10 µm
EPCAM
ACTA2
CD45
CD31
VIM

Week 13

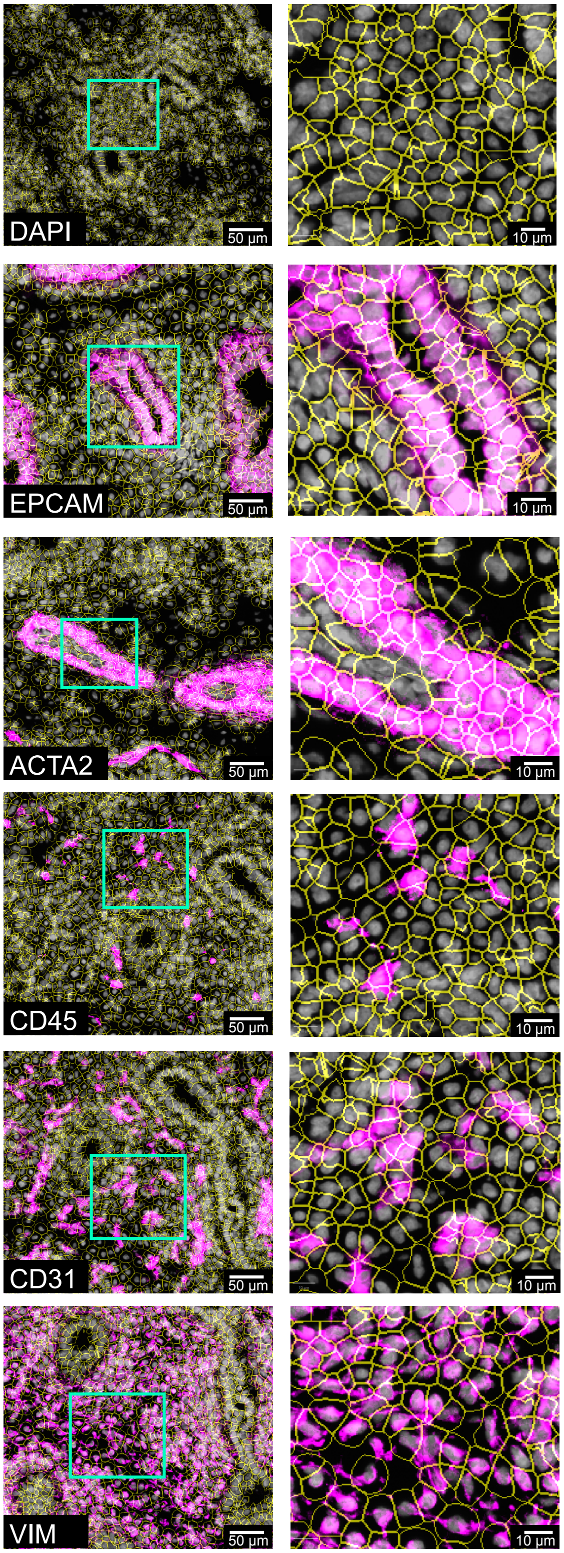

Supplementary Figure 3. Segmentation of the developing human lung tissue imaging data. DAPI and EPCAM images were used to create segmentation masks in PIPEX (https://github.com/CellProfiling/pipex.git) and the resulting .csv file included the intensities of each

marker, cell ID and coordinates of each segmented cell. For each week, the images in the left column show selected regions to demonstrate the fit of the segmentation mask for DAPI (nuclear marker), EPCAM (epithelial cells), ACTA2 (smooth muscle cells), CD45 (immune cells), CD31 (endothelial cells) and VIM (mesenchymal cells). The images in the right column display zoomed-in views within the green inset boxes. Scale bars correspond to 50 µm in the images on the left side, and to 10 µm in the images on right side for the zoomed-in green boxes. Images represent a single donor for each developmental week.

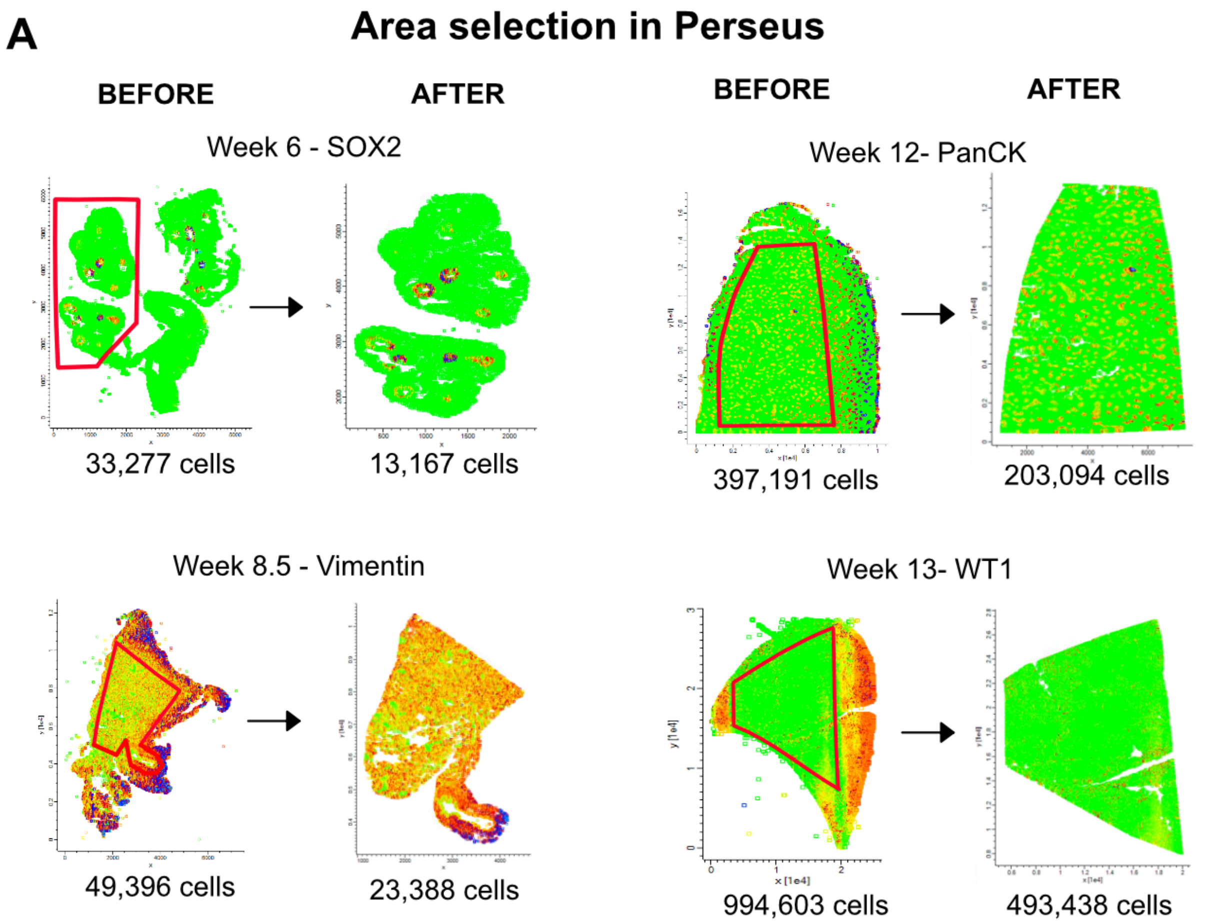

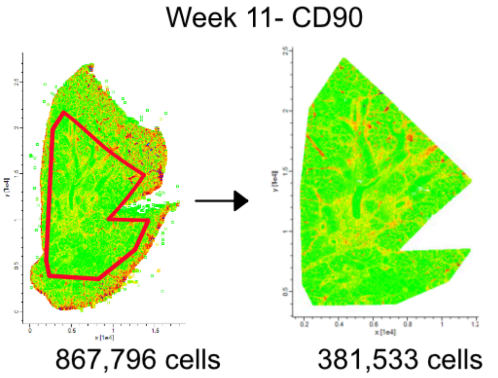

B

Number of cells after different stages of data processing

	6 pcw	8.5 pcw	11 pcw	12 pcw	13 pcw
Segmentation	34,930	643,84	978,277	493,588	1,341,495
DAPI & EPCAM filtration	33,277	49,396	867,796	397,191	994,603
Area selection	13167	27970	381,533	203,094	493,438
Artifact removal	11,713	24,837	350,208	179,615	355,198

C

Number of cells per mm^2

Week(pcw)	Number of cells	Area(mm^2)	Cells/mm^2
6	11,713	5.1	2,319
8.5	24,837	10.5	2,373
11	350,208	136.0	2,576
12	179,615	62.8	2,862
13	355,198	132.1	2,690
		Average	2,564

Supplementary Figure 4. Selection of developing human lung tissue regions for downstream analysis. A) Tissues were visualized back on the tissue plane as scatterplots according to the x and y coordinates of each cell. Using Perseus[2], different markers were selected for visualization of tissue at each week to examine the areas for potential imaging artifacts. On the scatter plots, green color indicates low expression and red color indicates high expression of the corresponding markers. For week 6 and 8.5, the areas not belonging to the lung tissue were excluded. For week 11, 12 and 13,

the edges of the tissue with artifacts were excluded. Red polygons indicate the regions selected in Perseus for the downstream analysis. Below each scatter plot, number of cells before and after area exclusion are indicated. **B)** Number of resulting cells after different stages of image and data processing: Segmentation, DAPI and EPCAM marker intensity filtration, area selection in Perseus and artifact removal after clustering. **C)** Calculated number of cells per unit area at different weeks, where the area corresponds to the selected polygons shown in panel **(A).**

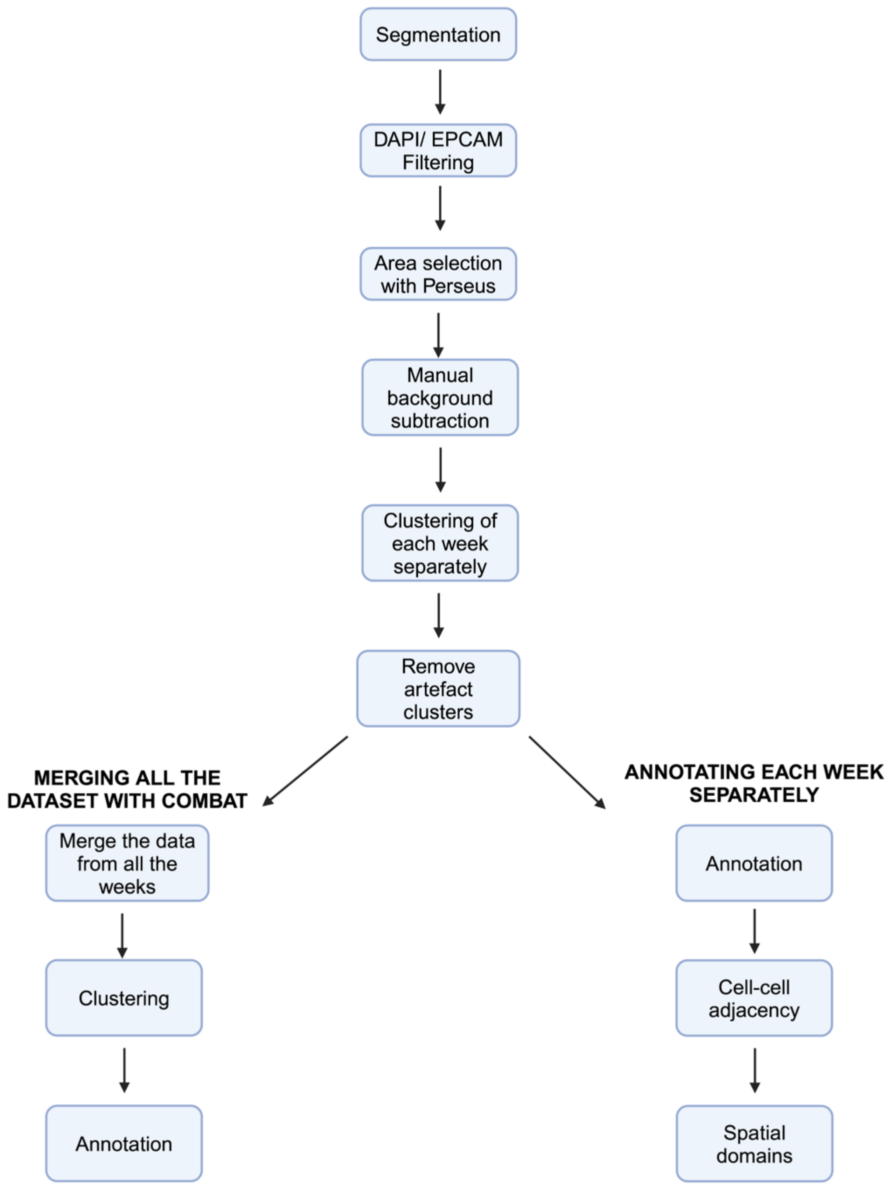

Supplementary Figure 5. Flowchart summarizing the main steps of imaging data processing and downstream analysis. Following image segmentation and intensity filtration for DAPI and EPCAM markers, artifact-free polygon areas were selected for each tissue using Perseus[2]. For each marker, background subtraction was performed. To identify and remove remaining artifact regions, data for each week was clustered separately and clusters annotated as artifacts were removed. Data from each week was then merged using ComBat[3] and the merged dataset was clustered and annotated to gain a global insight into the main cell types. Finally, clusters identified in each individual week were annotated for a more detailed analysis of cell types and subtypes and for a downstream analysis of cell type adjacency patterns and spatial domains in individual timepoints.

UMAP clusters before artifact removal

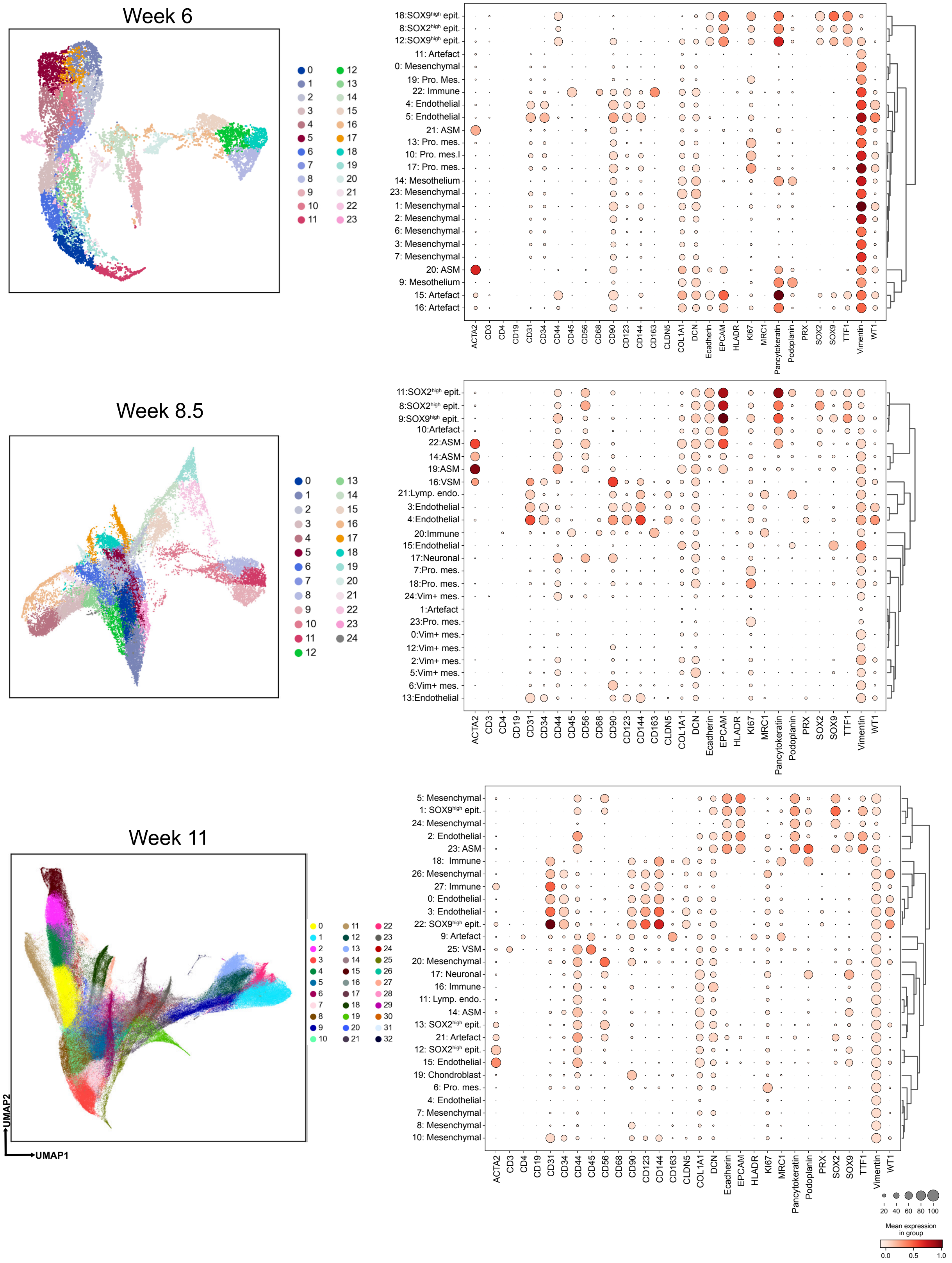

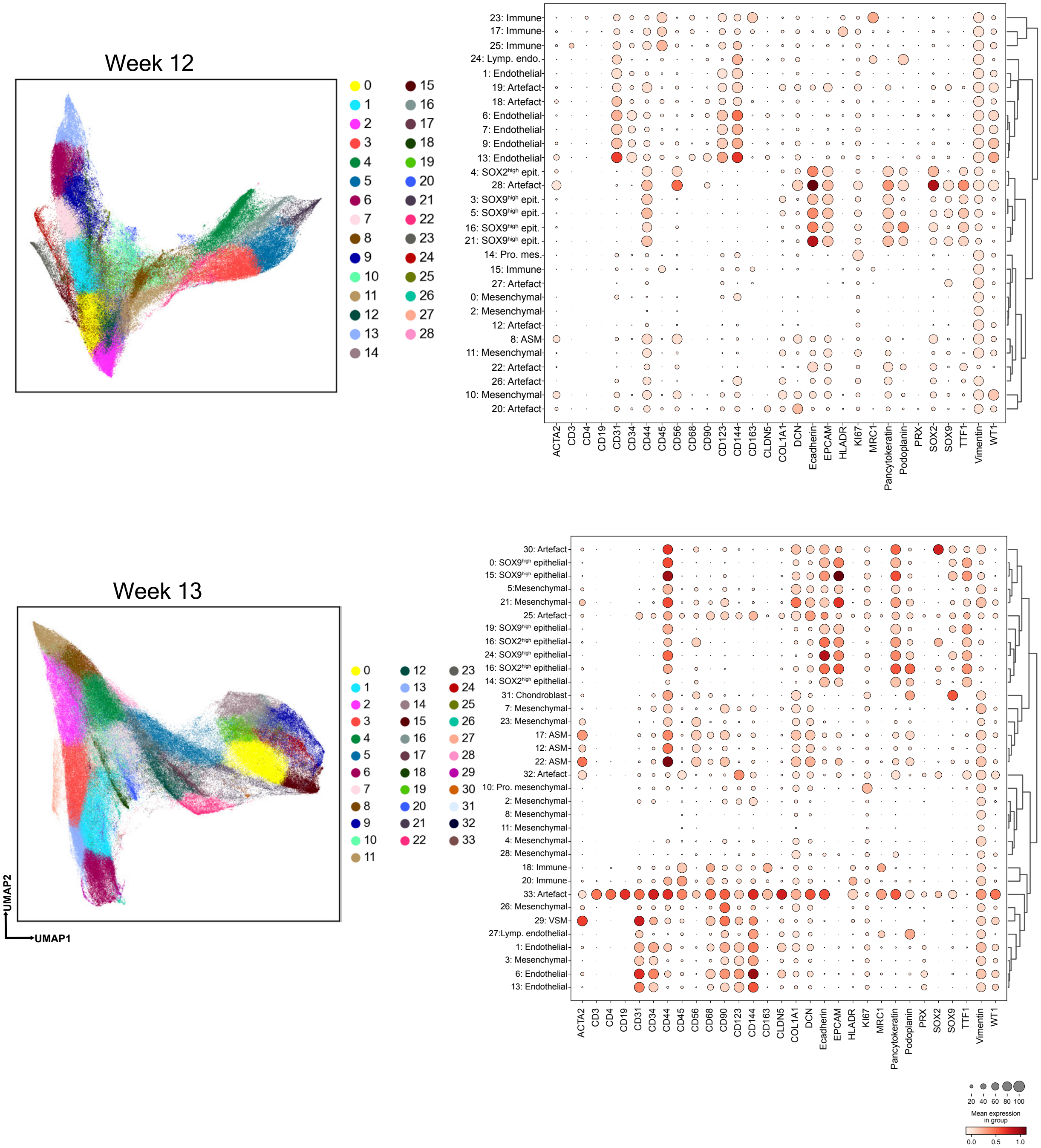

Supplementary Figure 6. Cluster annotations for developing human lung samples at different weeks before the removal of clusters with artifacts. UMAP plots on the left side show all clusters identified and dotplots show average marker expressions in these clusters, at each week. The size of the circles indicates the percentage of cells expressing the marker in each cluster, where dark red color indicates high and white color indicates low expression.

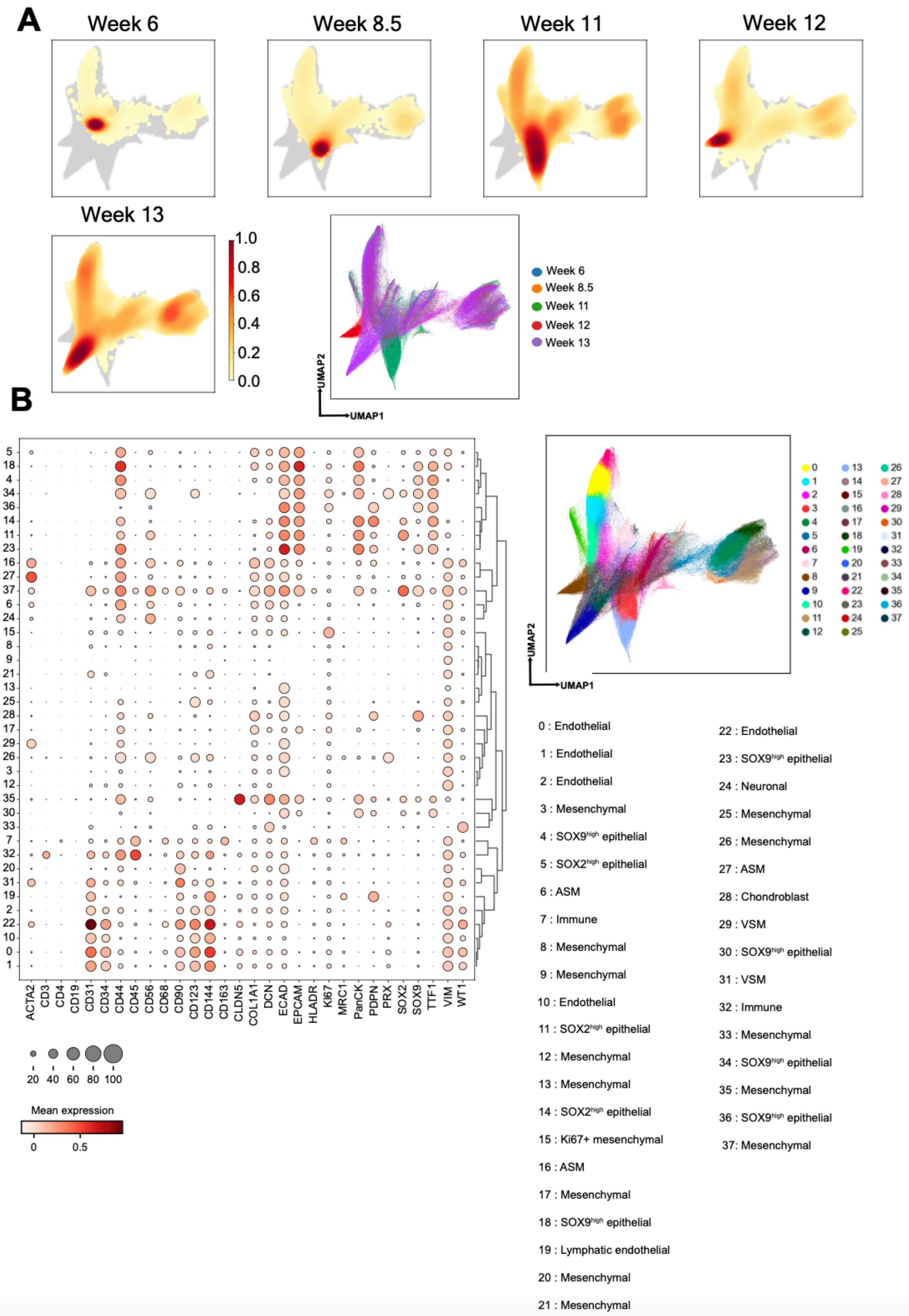
A
Week 6
Week 8.5
Week 11
Week 12
Week 13
1.0
0.8
0.6
0.4
0.2
0.0
Week 6
Week 8.5
Week 11
Week 12
Week 13
UMAP2
UMAP1
B
ACTA2 CD3 CD4 CD19 CD31 CD34 CD44 CD45 CD56 CD68 CD90 CD123 CD144 CD163 CLDN5 COL1A1 DCN ECAD EPCAM HLADR Ki67 MRC1 PanCK PDPN PRX SOX2 SOX9 TTF1 VIM WT1
5 18 4 34 36 14 11 23 16 27 37 6 24 15 8 9 21 13 25 28 17 29 26 3 12 35 30 33 7 32 20 31 19 2 22 10 0 1
20 40 60 80 100
Mean expression
0 0.5
UMAP2
UMAP1
0 : Endothelial
1 : Endothelial
2 : Endothelial
3 : Mesenchymal
4 : SOX9^{high} epithelial
5 : SOX2^{high} epithelial
6 : ASM
7 : Immune
8 : Mesenchymal
9 : Mesenchymal
10 : Endothelial
11 : SOX2^{high} epithelial
12 : Mesenchymal
13 : Mesenchymal
14 : SOX2^{high} epithelial
15 : Ki67+ mesenchymal
16 : ASM
17 : Mesenchymal
18 : SOX9^{high} epithelial
19 : Lymphatic endothelial
20 : Mesenchymal
21 : Mesenchymal
22 : Endothelial
23 : SOX9^{high} epithelial
24 : Neuronal
25 : Mesenchymal
26 : Mesenchymal
27 : ASM
28 : Chondroblast
29 : VSM
30 : SOX9^{high} epithelial
31 : VSM
32 : Immune
33 : Mesenchymal
34 : SOX9^{high} epithelial
35 : Mesenchymal
36 : SOX9^{high} epithelial
37: Mesenchymal

Supplementary Figure 7. Clustering of data merged from developing human lung samples at all analyzed weeks. A) Density plots embedded on UMAP plots show the distribution of clusters belonging to different weeks. High density is indicated with dark red color and low intensity is indicated with light yellow color. Below, UMAP plot of the merged data is shown where blue corresponds to data points from week 6, orange to week 8.5, green to week 11, red to week 12 and purple to week 13. **B)** Dotplot in the left panel shows the average marker expression in merged data. The size of the circles indicates the percentage of cells expressing the marker in each cluster, where dark red color indicates high and white color indicates low expression. In the lower panel, annotations of each cluster of the merged data are provided.

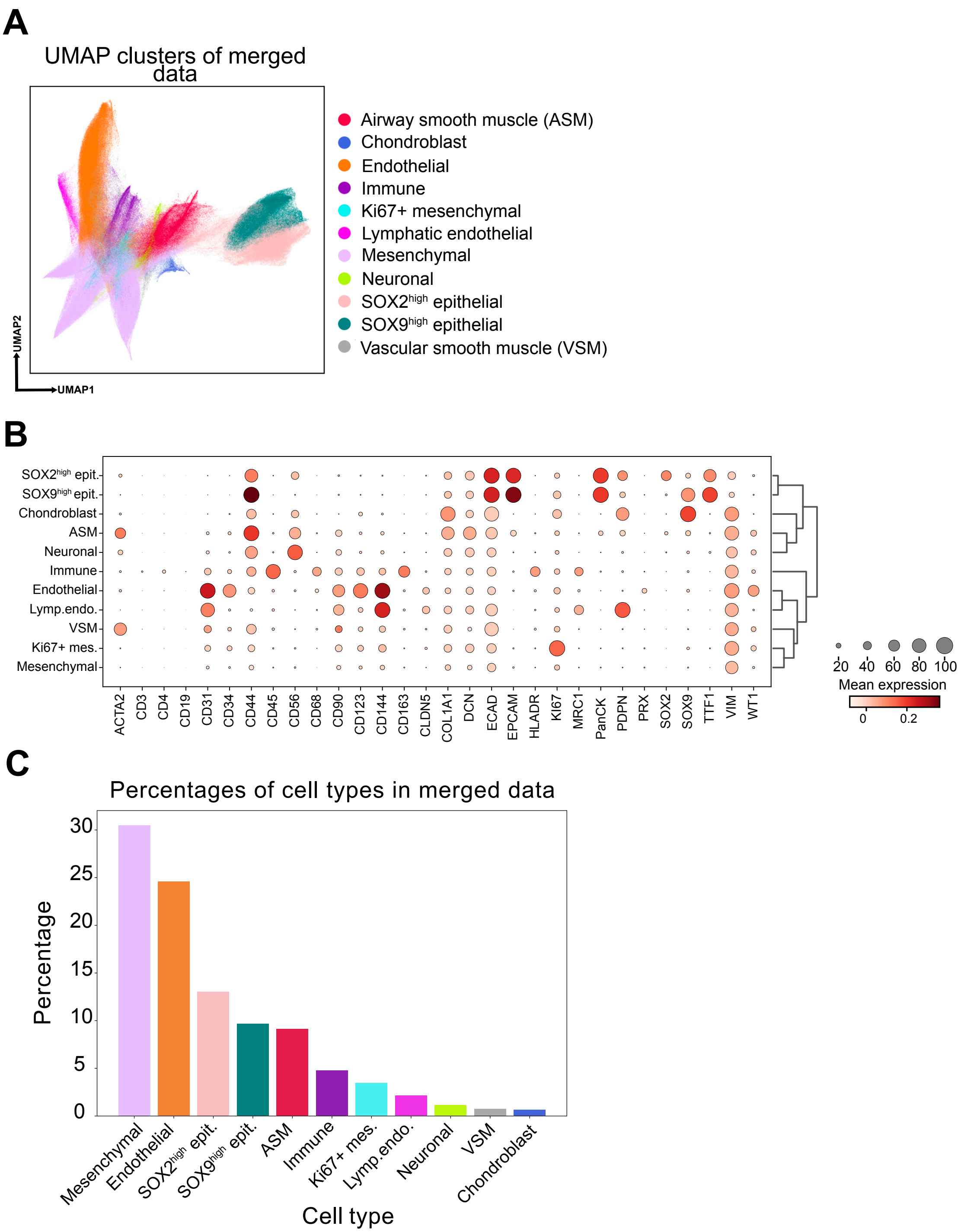

Supplementary Figure 8. Clustering of merged human lung data across all developmental weeks. A) Annotated cell type clusters visualized on the UMAP plot. The x-axis shows UMAP1 and the y-axis shows UMAP2. **B)** Dot plot shows the average marker expression across annotated cell type clusters. The size of the circles indicates the fraction of cells expressing the marker in each cluster. Dark red color indicates high and white color indicates low expression. **C)** Percentage of

different cell types annotated in the merged data. Source data for this figure is provided within the Source Data file.

UMAP clusters after artifact removal

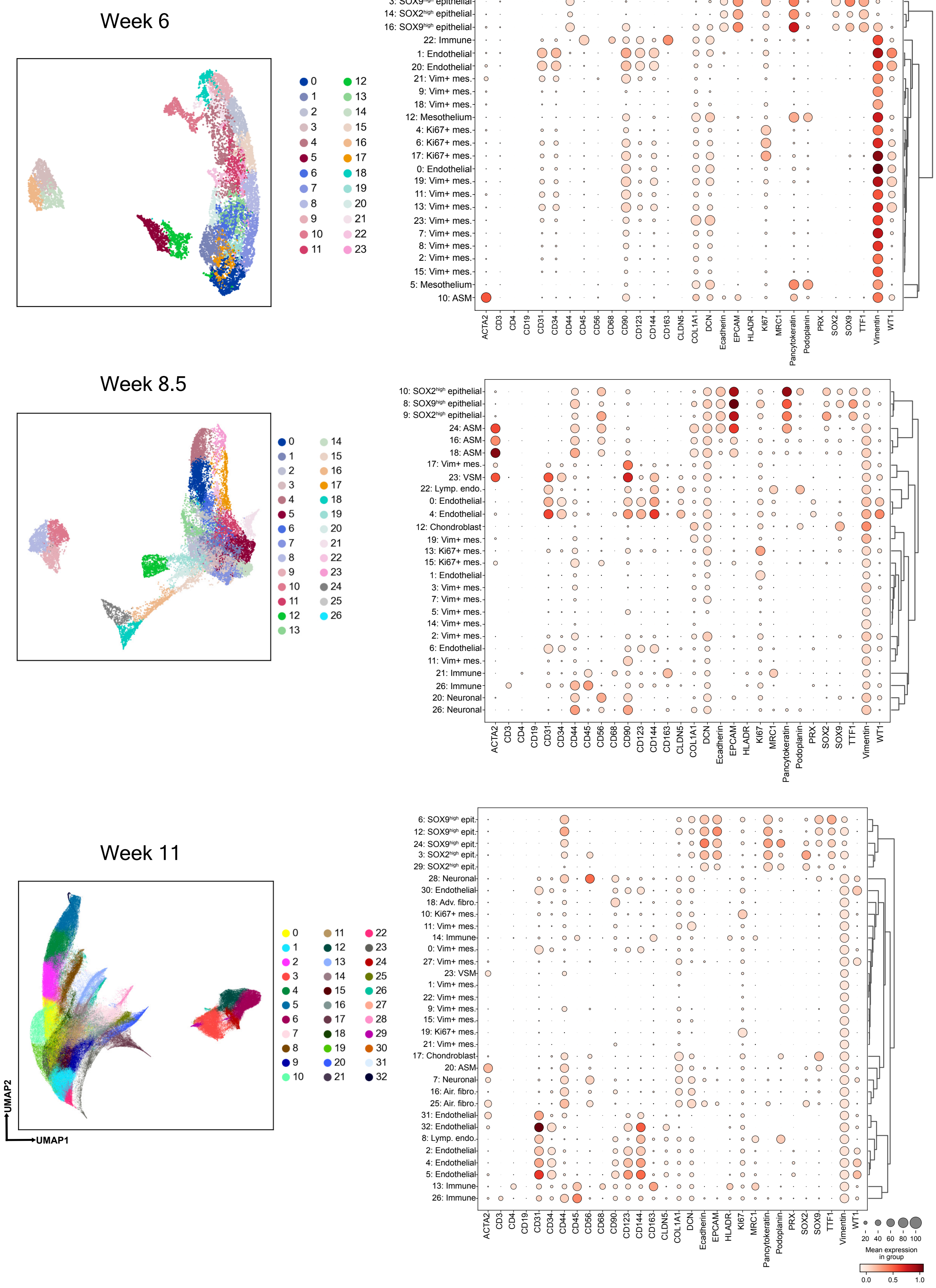

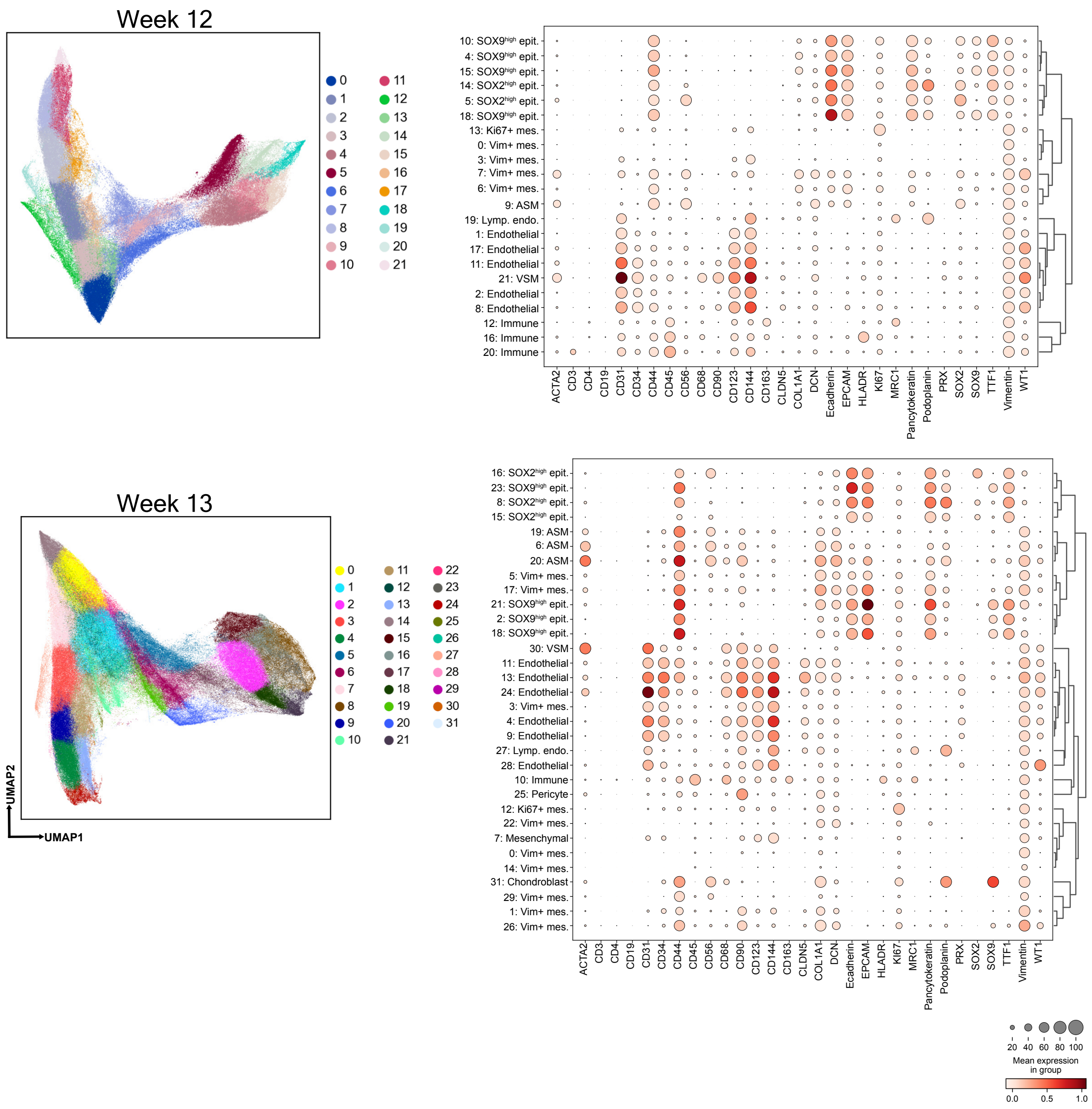

Supplementary Figure 9. Cluster annotations for developing human lung samples at different weeks after removal of clusters with artifacts. On the left panel, all the clusters after artifact removal are shown on the UMAP plots at each week. Dotplots to the right show average marker expression in each cluster. The size of the circles indicates the fraction of cells expressing the marker in each cluster, where dark red color indicates high and white color indicates low expression.

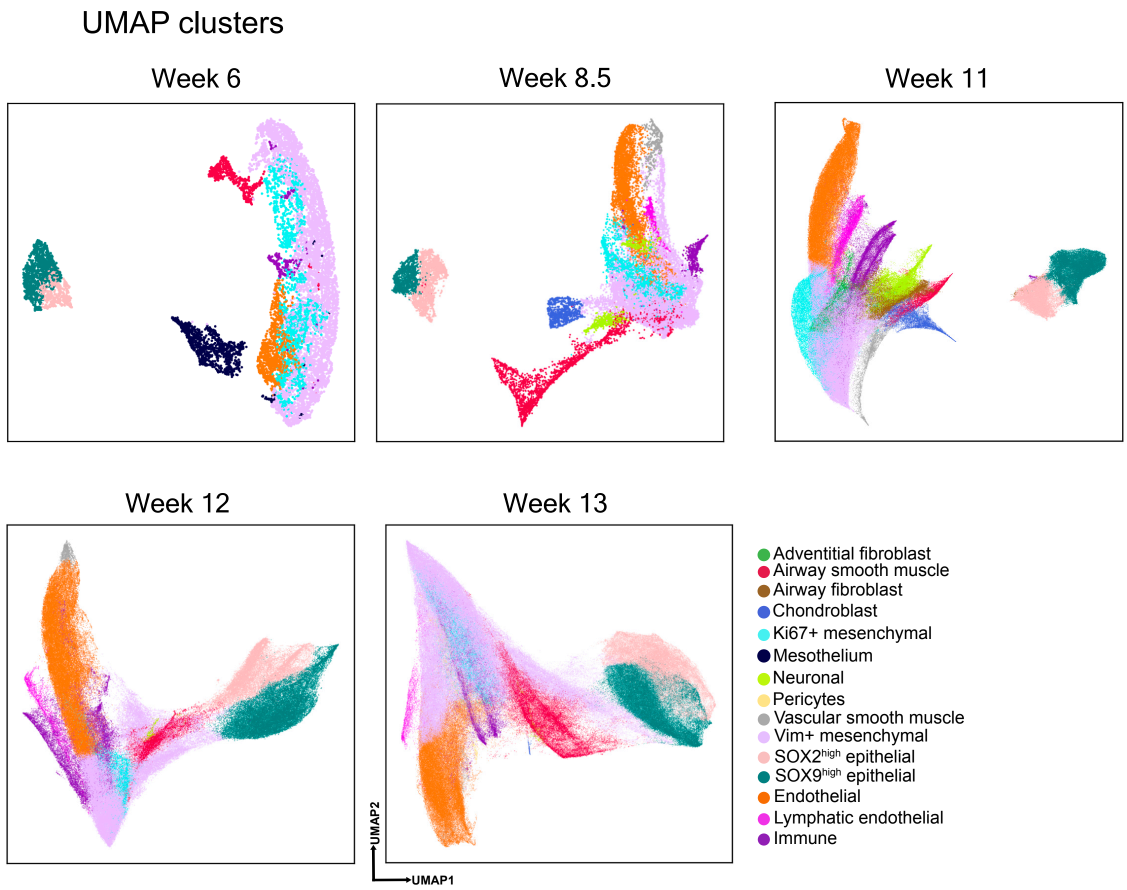

Supplementary Figure 10. Cluster annotations for developing human lung samples at different weeks. Clusters belonging to the same cell type were merged and plotted on UMAP embedding. Annotations of all identified clusters, represented with different colors, are provided.

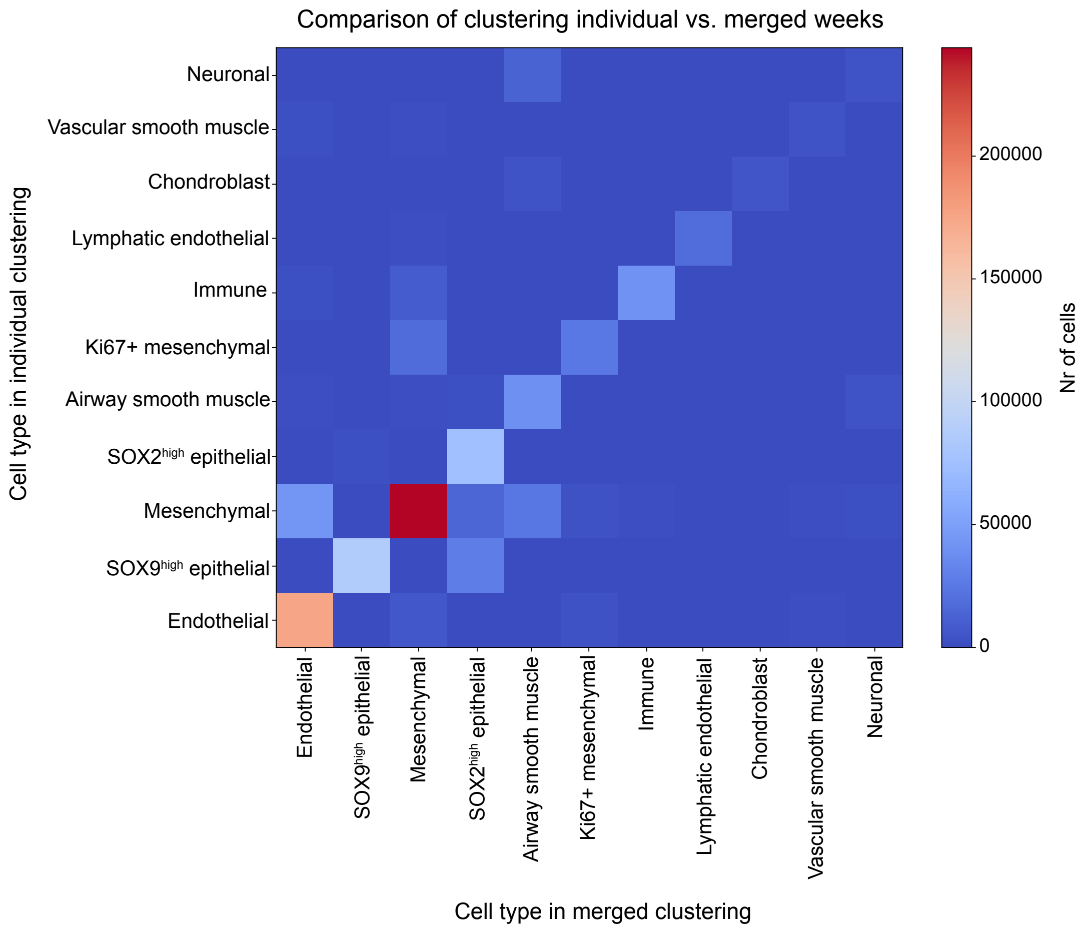

Supplementary Figure 11. Comparison of the assigned cell type annotations in developing human lung tissue as the outcome of clustering individual versus merged timepoints. The heatmap summarizes the count of cells assigned to one of the eleven cell types for individual and merged clustering of timepoints. While the majority (78%) of the cell type assignments were consistent, discrepancies existed in the assignment of certain cell types such as SOX2^{high} epithelial cells, immune cells and airway smooth muscle cells.

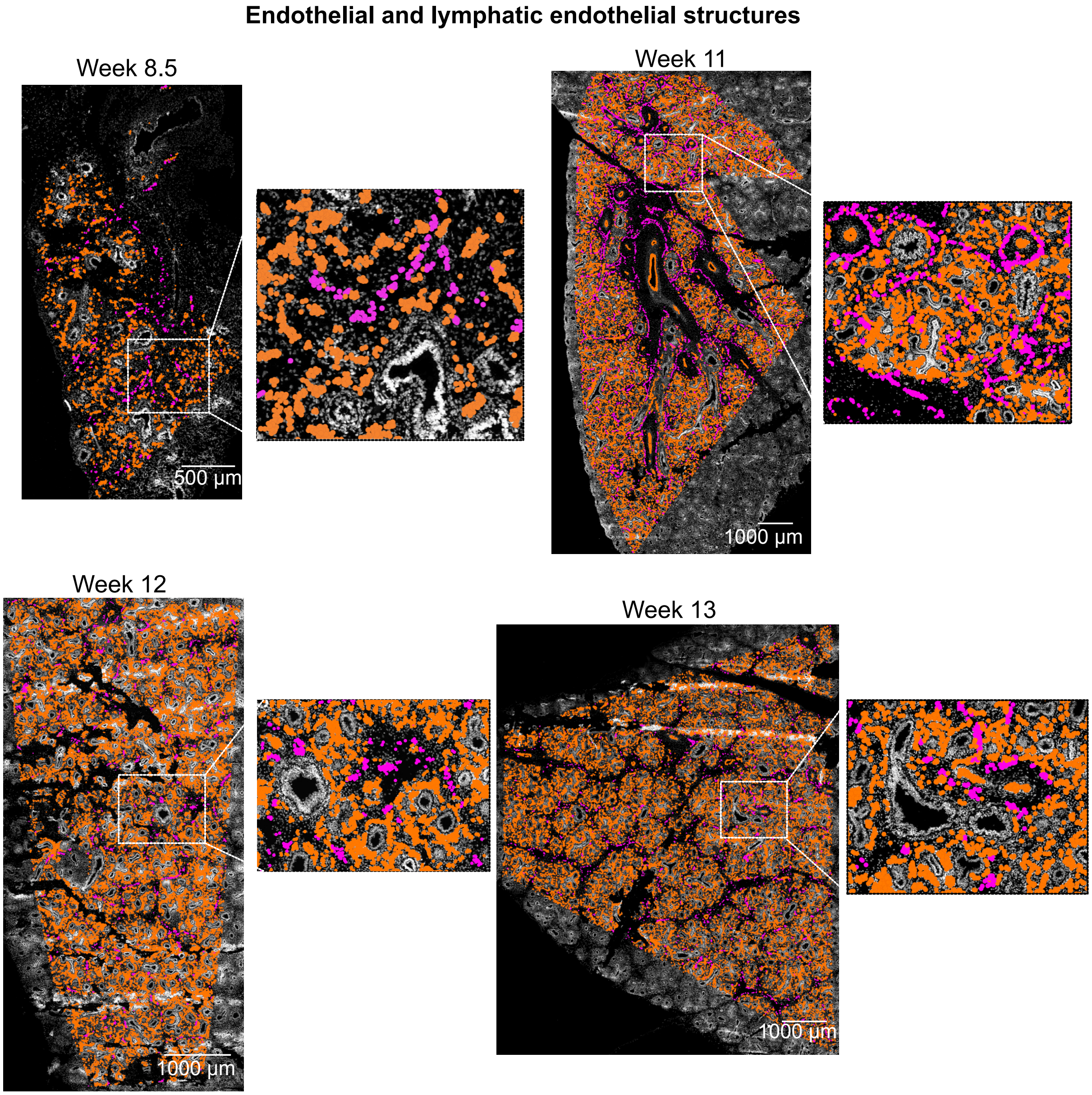

Supplementary Figure 12. Endothelial and lymphatic endothelial structures in developing human lung. Clusters annotated as "endothelial" (dark orange) and "lymphatic endothelial "(magenta) overlaid on DAPI channel images for each week. Images represent a single donor for each developmental week. Scale bars correspond to 500 µm for week 8.5, and 1,000 µm for weeks 11,12 and 13.

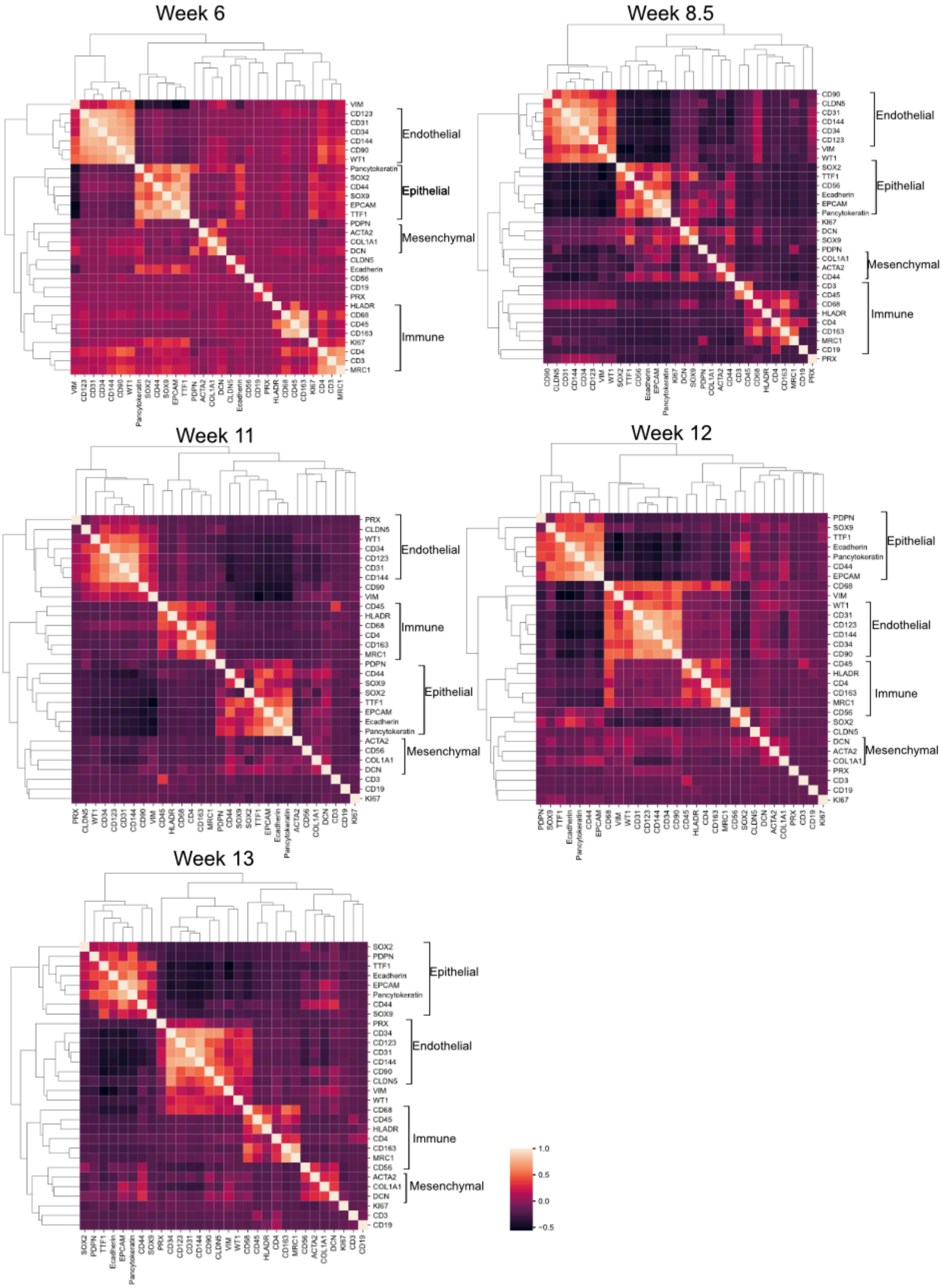
Week 6
VIM
CD123
CD31
CD34
CD144
CD90
WT1
Pancytokeratin
SOX2
CD44
SOX9
EPCAM
TTF1
PDPN
ACTA2
COL1A1
DCN
CLDN5
Ecadherin
CD56
CD19
PRX
HLADR
CD68
CD45
CD163
KI67
CD4
CD3
MRC1
Endothelial
Epithelial
Mesenchymal
Immune
Week 8.5
CD90
CLDN5
CD31
CD144
CD34
CD123
VIM
WT1
SOX2
TTF1
CD56
Ecadherin
EPCAM
Pancytokeratin
KI67
DCN
SOX9
PDPN
COL1A1
ACTA2
CD44
CD3
CD45
CD68
HLADR
CD4
CD163
MRC1
CD19
PRX
Endothelial
Epithelial
Mesenchymal
Immune
Week 11
PRX
CLDN5
WT1
CD34
CD123
CD31
CD144
CD90
VIM
CD45
HLADR
CD68
CD4
CD163
MRC1
PDPN
CD44
SOX9
SOX2
TTF1
EPCAM
Ecadherin
Pancytokeratin
ACTA2
CD56
COL1A1
DCN
CD3
CD19
KI67
Endothelial
Immune
Epithelial
Mesenchymal
Week 12
PDPN
SOX9
TTF1
Ecadherin
Pancytokeratin
CD44
EPCAM
CD68
VIM
WT1
CD31
CD123
CD144
CD34
CD90
CD45
HLADR
CD4
CD163
MRC1
CD56
SOX2
CLDN5
DCN
ACTA2
COL1A1
PRX
CD3
CD19
KI67
Epithelial
Endothelial
Immune
Mesenchymal
Week 13
SOX2
PDPN
TTF1
Ecadherin
EPCAM
Pancytokeratin
CD44
SOX9
PRX
CD34
CD123
CD31
CD144
CD90
CLDN5
VIM
WT1
CD68
CD45
HLADR
CD4
CD163
MRC1
CD56
ACTA2
COL1A1
DCN
KI67
CD3
CD19
Epithelial
Endothelial
Immune
Mesenchymal
1.0
0.5
0.0
-0.5

Supplementary Figure 13. Marker correlations in developing human lung samples at different weeks. Pearson correlation coefficients are plotted in heatmaps, where Pearson's r=1 corresponds to white and r=-0.5 corresponds to black.

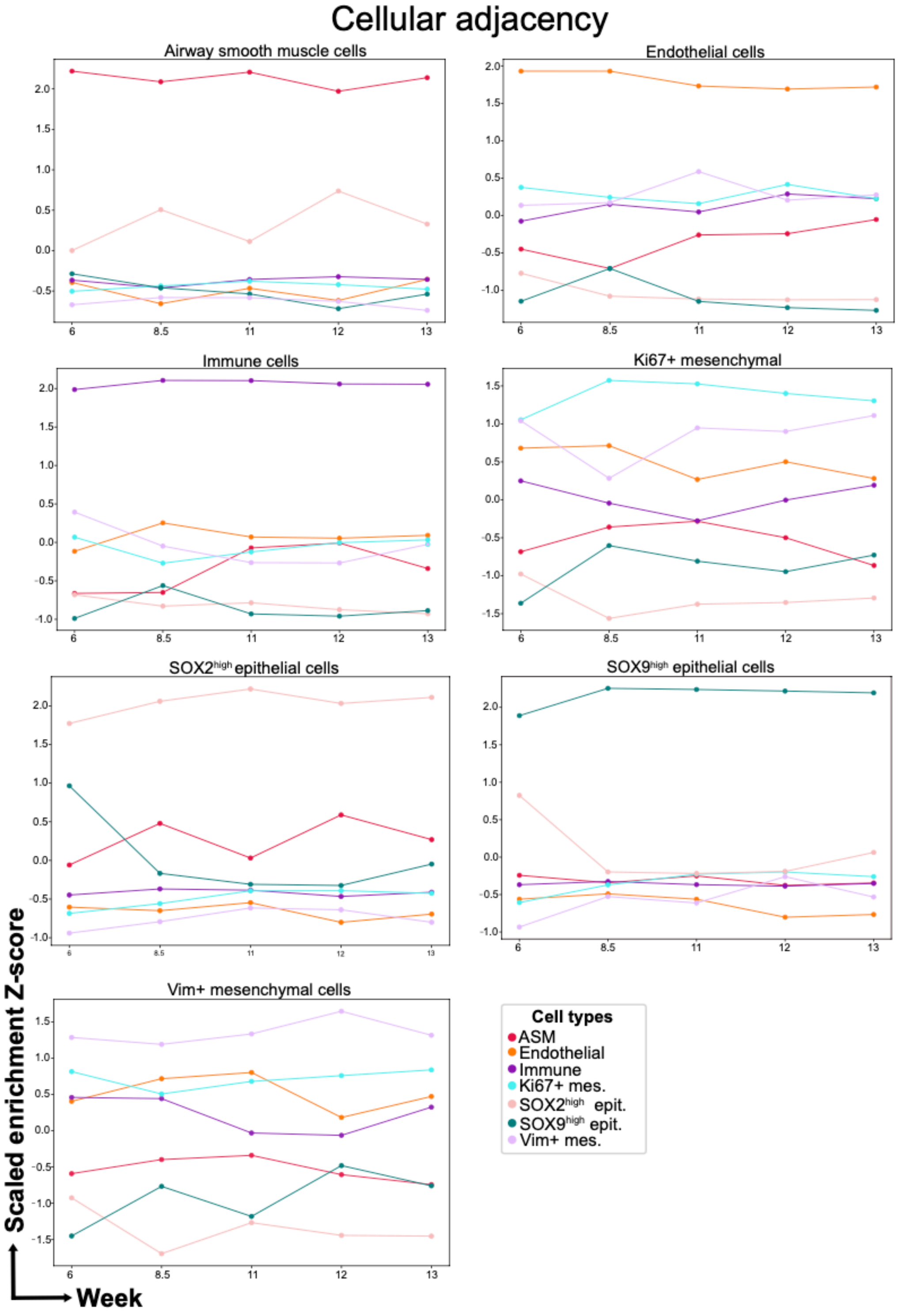

Supplementary Figure 14. Temporal changes of scaled neighborhood enrichment Z-scores for the main cell types of developing human lung. Each lineplot summarizes the temporal change of scaled enrichment Z-score for each main cell type for neighboring their own, or the rest of the other cell types, where each line represents one of the seven main cell types in developing human lung. Source data for this figure is provided within the Source Data file.

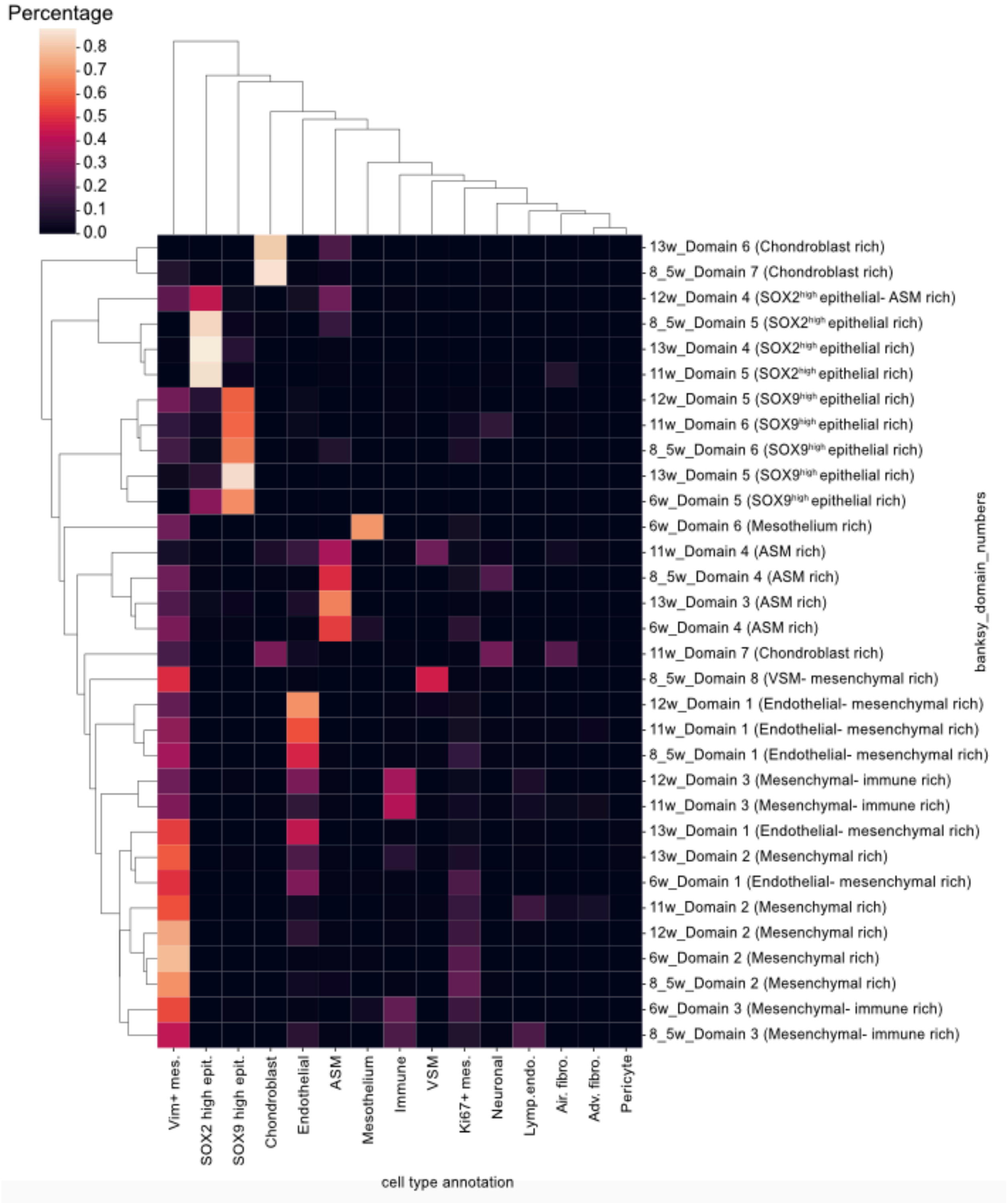

Supplementary Figure 15. Hierarchical clustering of the cell type composition across all individual spatial domains identified in the developing human lung at different weeks. The color scale indicates the percentage of each cell type within a domain.

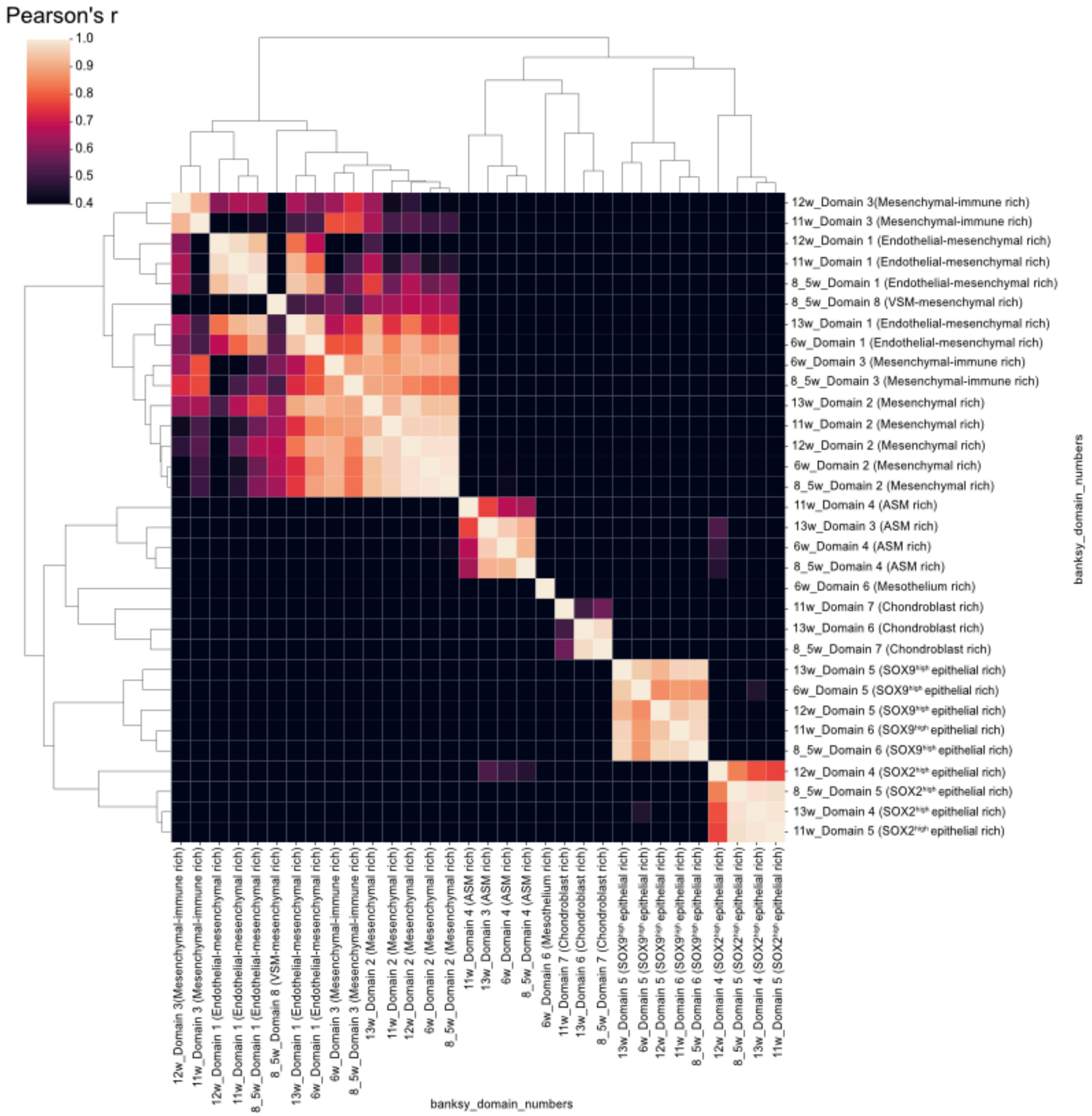

Supplementary Figure 16. Hierarchical clustering of the correlation among all the individual spatial domains identified in the developing human lung at different weeks. The color scale indicates Pearson correlation coefficient r.

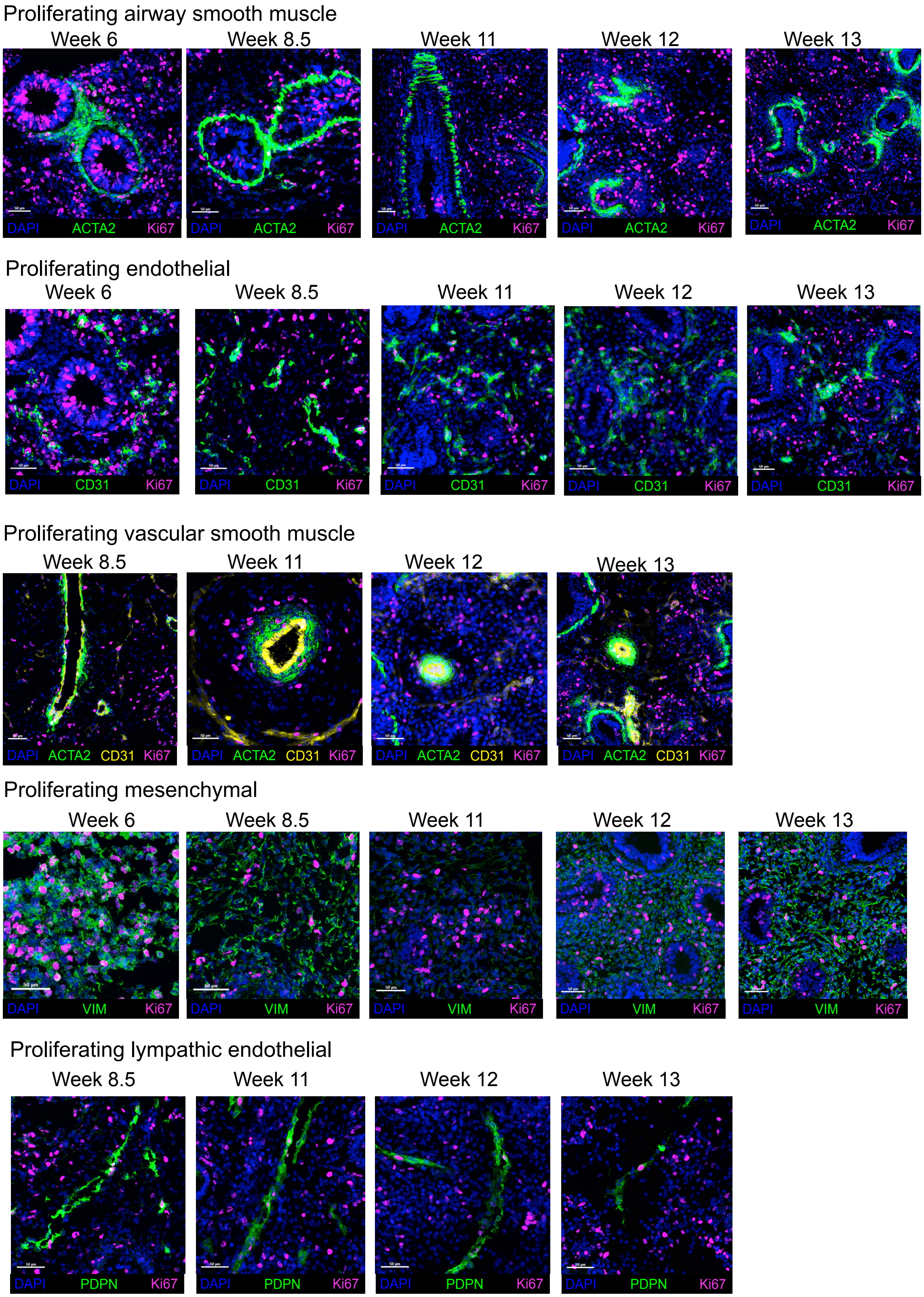
Proliferating airway smooth muscle
Week 6
Week 8.5
Week 11
Week 12
Week 13
DAPI ACTA2 Ki67
Proliferating endothelial
Week 6
Week 8.5
Week 11
Week 12
Week 13
DAPI CD31 Ki67
Proliferating vascular smooth muscle
Week 8.5
Week 11
Week 12
Week 13
DAPI ACTA2 CD31 Ki67
Proliferating mesenchymal
Week 6
Week 8.5
Week 11
Week 12
Week 13
DAPI VIM Ki67
Proliferating lymphatic endothelial
Week 8.5
Week 11
Week 12
Week 13
DAPI PDPN Ki67

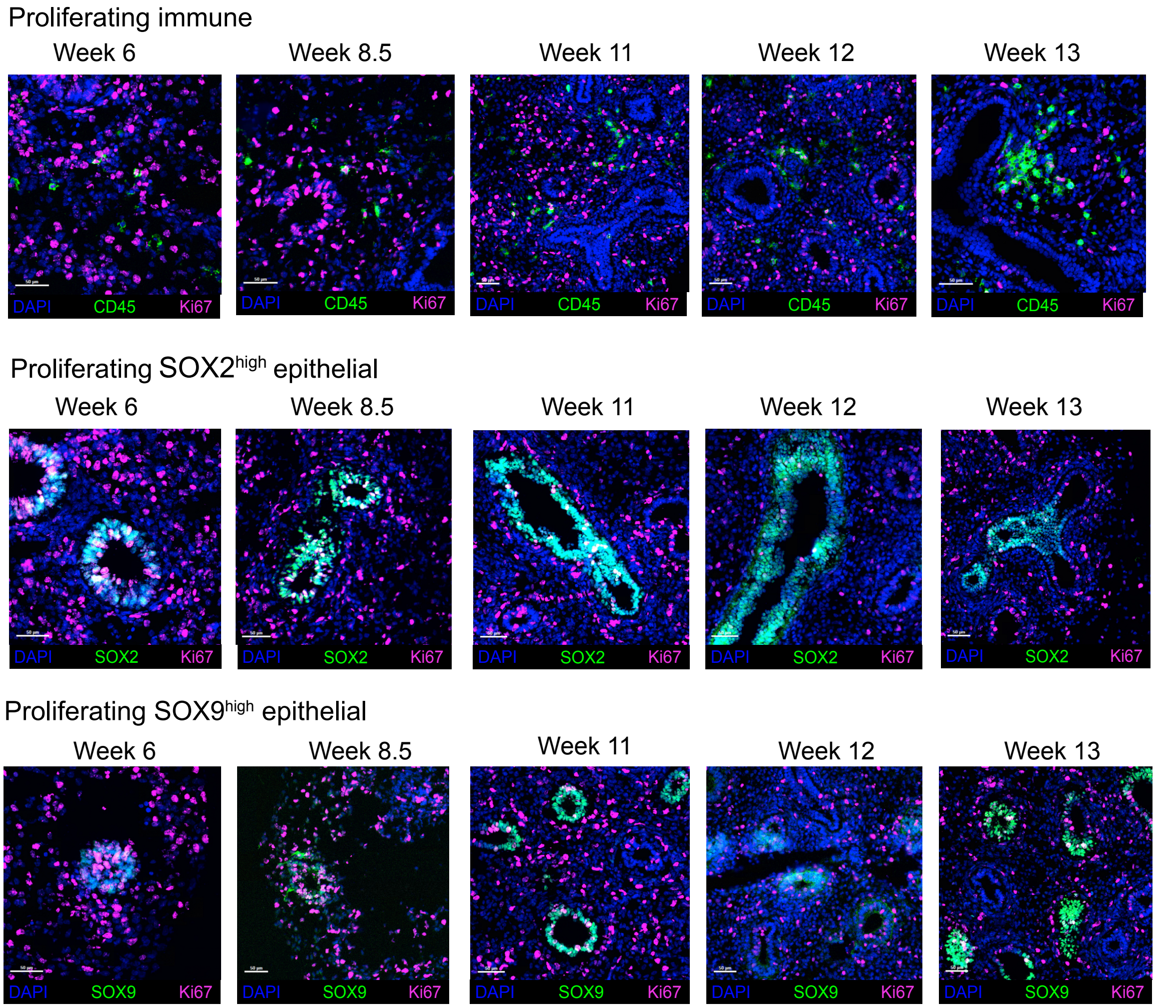

Supplementary Figure 17. Expression patterns of Ki67 across the main cell types of the human developing lung. Expression of Ki67 (magenta) was overlaid with expression of ACTA2 (green) for airway smooth muscle cells, CD31 (green) for endothelial cells, ACTA2 (green) and CD31 (yellow) for vascular smooth muscle cells, VIM (green) for mesenchymal cells, PDPN (green) for lymphatic endothelial cells, CD45 (green) for immune cells, SOX2 (green) for SOX2^{high} epithelial cells and SOX9 (green) for SOX9^{high} epithelial cells. Images represent a single donor for each developmental week. Scale bars correspond to 50 µm.

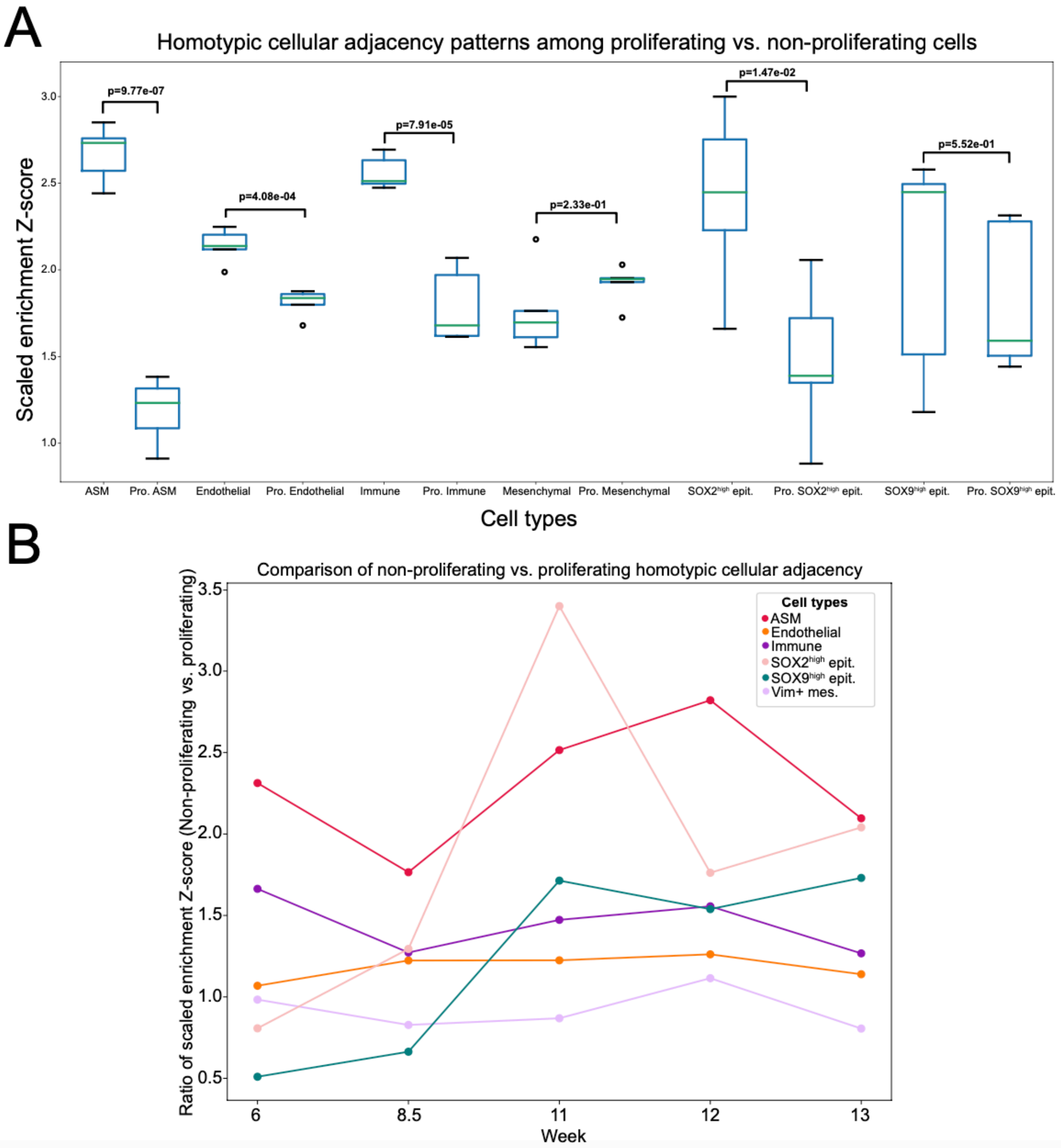

Supplementary Figure 18. Comparison of homotypic cellular adjacency patterns among the non-proliferating cell types and their proliferating counterparts in the developing human lung. A) Boxplots summarizing the scaled neighborhood enrichment Z-scores of six different cell types for its own cell type, where the neighborhood enrichment score for each non-proliferating and proliferating cell type across all the five developmental time points was compared in a two-sided Student's t-test. Source data for this figure is provided within the Source Data file. **B)** Lineplots summarizing the ratio of scaled enrichment Z-scores in non-proliferating versus proliferating counterparts of each cell type across the five developmental timepoints, where each line represents a different cell type. Source data for this figure is provided within the Source Data file.

A

Week 6

Original Ann.	Simplified Ann.
Airway smooth muscle	Airway smooth muscle
Endothelial	Endothelial
Ki67+ mesenchymal	Mesenchymal
Mesothelium	Mesenchymal
Immune	Immune
SOX2^{high} epithelial	SOX2^{high} epithelial
SOX9^{high} epithelial	SOX9^{high} epithelial
Vim+ mesenchymal	Mesenchymal

Week 8.5

Original Ann.	Simplified Ann.
Airway smooth muscle	Airway smooth muscle
Endothelial	Endothelial
KI67+ mesenchymal	Mesenchymal
Lymphatic endothelial	Lymphatic endothelial
Immune	Immune
SOX2^{high} epithelial	SOX2^{high} epithelial
SOX9^{high} epithelial	SOX9^{high} epithelial
Vascular smooth muscle	Vascular smooth muscle
Vim+ mesenchymal	Mesenchymal
Neuronal	Mesenchymal
Chondroblasts	Mesenchymal

Week 11

Original Ann.	Simplified Ann.
Adventitial fibroblast	Mesenchymal
Airway fibroblast	Mesenchymal
Airway smooth muscle	Airway smooth muscle
Chondroblast	Mesenchymal
Endothelial	Endothelial
Ki67+ mesenchymal	Mesenchymal
Lymphatic endothelial	Lymphatic endothelial
Immune	Immune
Neuronal	Mesenchymal
SOX2^{high} epithelial	SOX2^{high} epithelial
SOX9^{high} epithelial	SOX9^{high} epithelial
Vascular smooth muscle	Vascular smooth muscle
Vim+ mesenchymal	Mesenchymal

Week 12

Original Ann.	Simplified Ann.
Airway smooth muscle	Airway smooth muscle
Endothelial	Endothelial
Ki67+ mesenchymal	Mesenchymal
Lymphatic endothelial	Lymphatic endothelial
Immune	Immune
SOX2^{high} epithelial	SOX2^{high} epithelial
SOX9^{high} epithelial	SOX9^{high} epithelial
Vascular smooth muscle	Vascular smooth muscle
Vim+ mesenchymal	Mesenchymal
Neuronal	Mesenchymal

Week 13

Original Ann.	Simplified Ann.
Airway smooth muscle	Airway smooth muscle
Chondroblast	Mesenchymal
Endothelial	Endothelial
Ki67+ mesenchymal	Mesenchymal
Lymphatic endothelial	Lymphatic endothelial
Neuronal	Mesenchymal
Immune	Immune
Pericytes	Mesenchymal
SOX2^{high} epithelial	SOX2^{high} epithelial
SOX9^{high} epithelial	SOX9^{high} epithelial
Vascular smooth muscle	Vascular smooth muscle
Vim+ mesenchymal	Mesenchymal

B

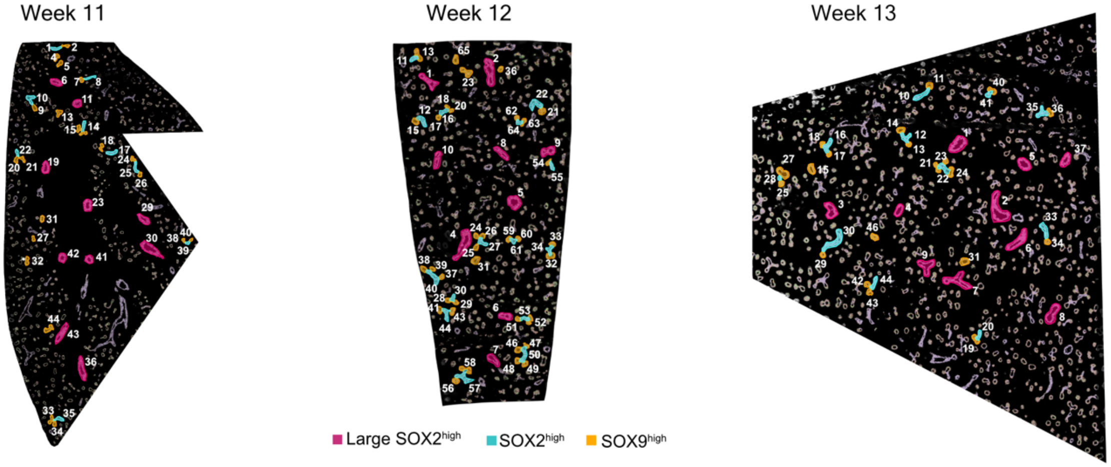

C

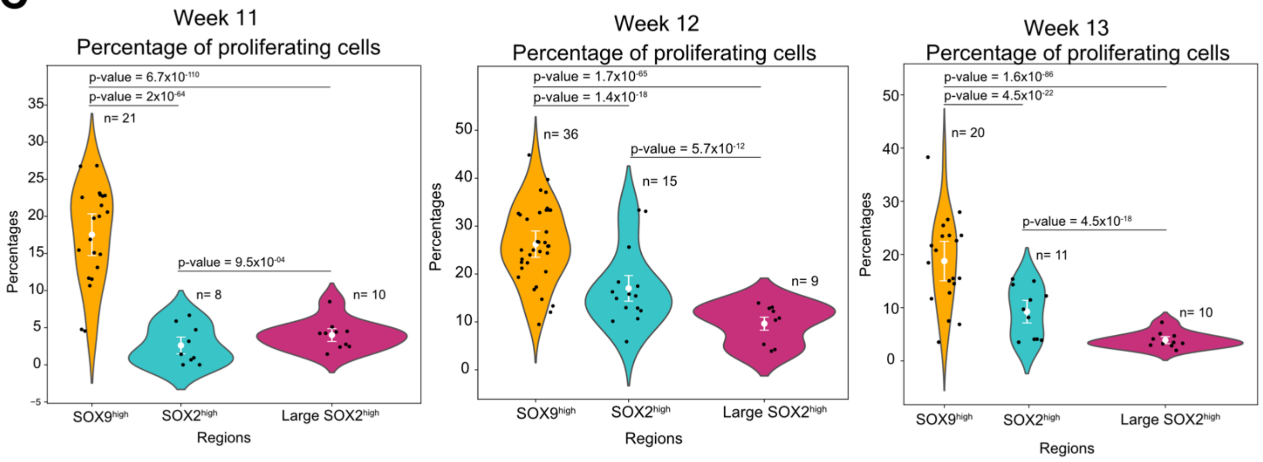

Supplementary Figure 19. Cell-type specific proliferation patterns in the developing human lung. A) Simplified annotations used for the proliferation analysis. The first column shows the original cell type annotation and the second column shows the simplified annotations for the proliferation analysis. **B)** The selected regions for analysis and comparison of proliferation patterns in relatively smaller $SOX2^{high}$ (cyan), relatively larger $SOX2^{high}$ (magenta) and $SOX9^{high}$ (orange) epithelial regions. ID of each identified and used region for analysis is stated on the tissue images. **C)** Violin plots displaying the distribution of the fraction of proliferating cells in relatively smaller $SOX2^{high}$ (cyan), relatively larger $SOX2^{high}$ (magenta) and $SOX9^{high}$ (orange) epithelial regions at each week. The x-axis represents the three different airway structures, and the y-axis indicates the percentage of proliferating cells in each of the (n) number of manually selected regions represented as a dot, and the error bar represents mean±2×SEM (standard error of the mean). The statistical significance of the differences in proliferation degree are summarized with a two-sided Fisher's exact test p-values. Source data for this figure is provided within the Source Data file.

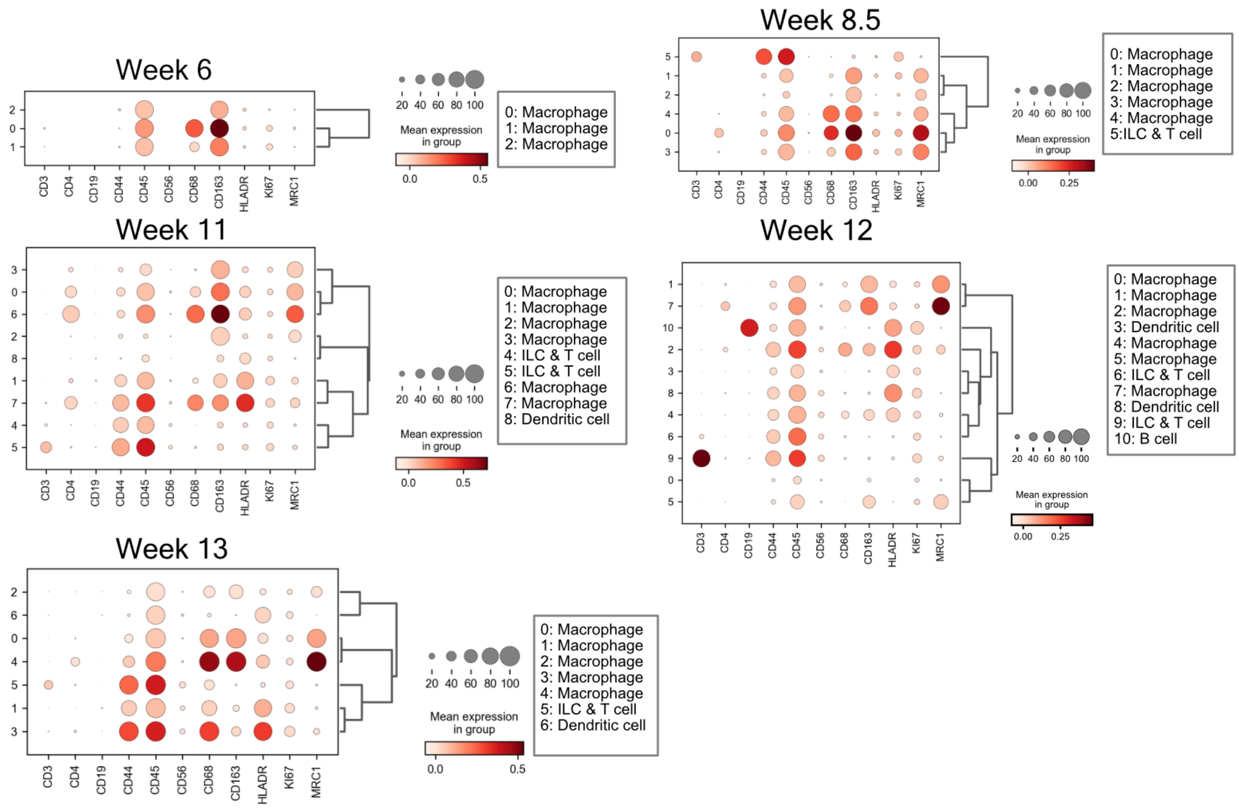

Supplementary Figure 20. Subclustering of the major immune cell clusters identified in developing human lung. The major immune cluster identified at each week was re-clustered and annotated based on the marker expressions in the dot plots. The size of the circles indicates the percentage of cells expressing the marker in each cluster, where dark red color indicates high and white color indicates low expression.

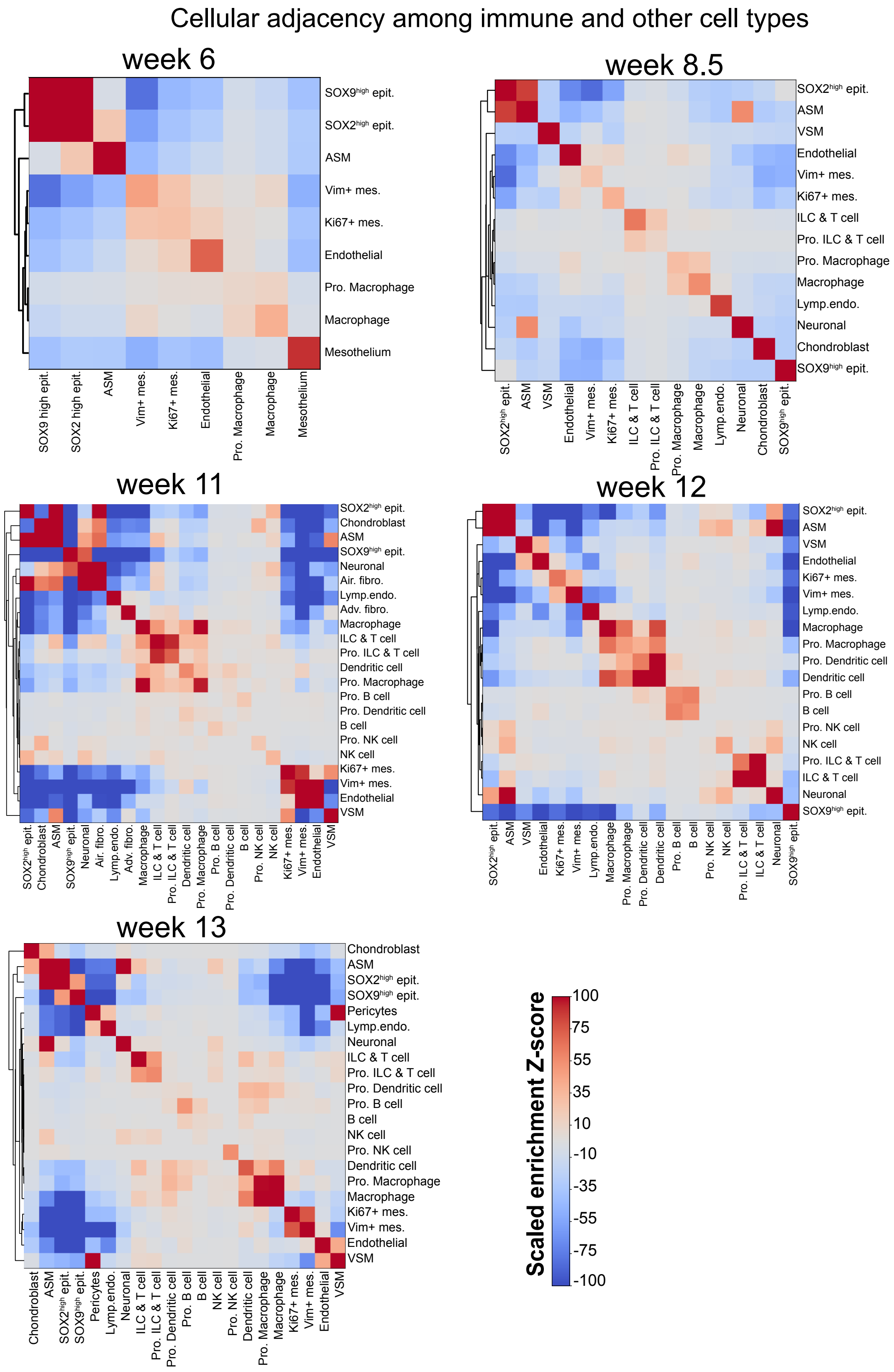

Supplementary Figure 21. Heatmaps with dendrograms summarizing enrichment of cellular adjacency patterns among immune and other cell types based on the scaled Z-scores. Dark red

color indicates cellular adjacency patterns with high enrichment Z-score and dark blue color indicates cellular adjacency patterns with low enrichment Z-score.

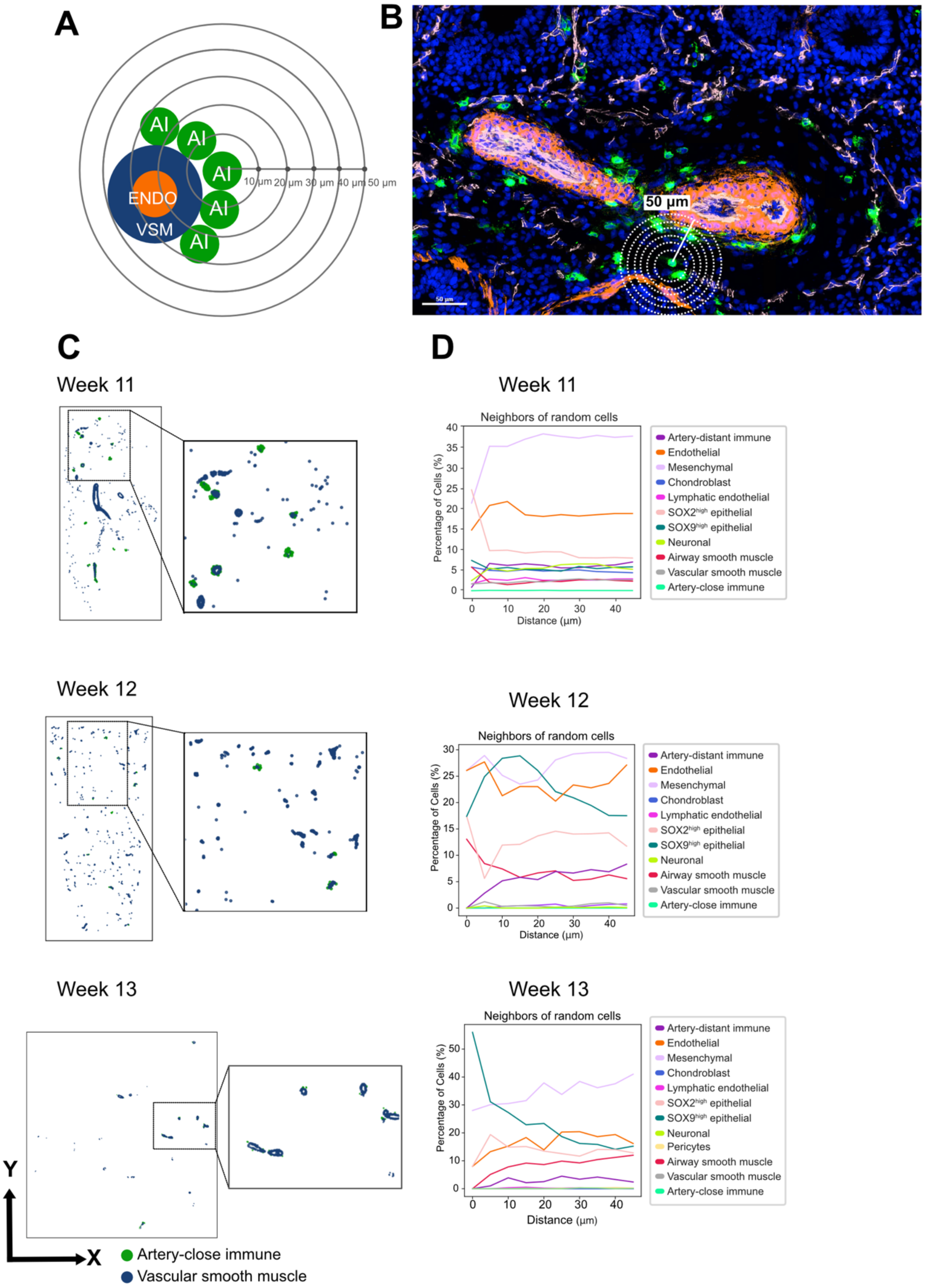
A
AI
ENDO
VSM
10 μm 20 μm 30 μm 40 μm 50 μm
B
50 μm
C
Week 11
Week 12
Week 13
Y
X
Artery-close immune
Vascular smooth muscle
D
Week 11
Neighbors of random cells
Percentage of Cells (%)
Distance (μm)
Artery-distant immune
Endothelial
Mesenchymal
Chondroblast
Lymphatic endothelial
SOX2high epithelial
SOX9high epithelial
Neuronal
Airway smooth muscle
Vascular smooth muscle
Artery-close immune
Week 12
Neighbors of random cells
Week 13
Neighbors of random cells
Pericytes

Supplementary Figure 22. Neighborhood cellular composition analysis of immune cells encircling the arteries. A) Scheme describing the neighborhood cellular composition analysis of the artery-close immune cells (AI) encircling vascular smooth muscle cells (VSM) and endothelial cells (ENDO). **B)** Snapshots of immune cells encircling arteries and a representation of the analyzed cellular composition for a radius of 50 µm around each detected artery-close immune cell. DAPI in blue, CD45 (immune marker) in green, ACTA2 (vascular smooth muscle marker) in orange and CD144 (endothelial marker) in pink. Image represent a single donor. Scale bar corresponds to 50 µm. **C)** The xy-scatter plots show the location of immune cells detected around arteries and vascular smooth muscle cells. **D)** Line plots summarizing the cell type and count of neighbors of randomly selected cells within a radius of 50 µm for weeks 11,12 and 13. The x-axis represents the distance in µm and the y-axis represents the fraction of cells. Source data for this figure is provided within the Source Data file.

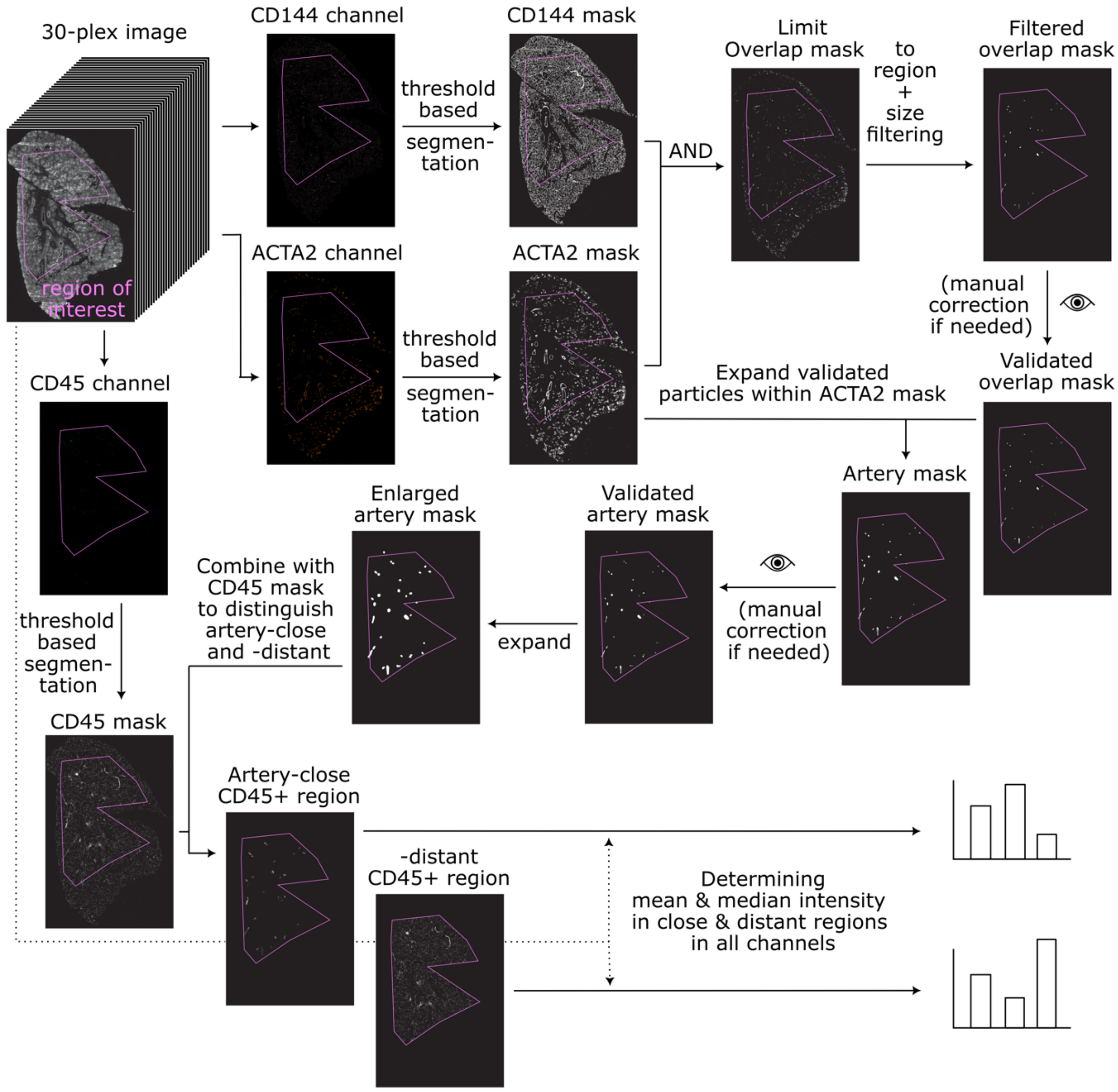

Supplementary Figure 23. Image analysis workflow to validate marker expression patterns of artery-close and -distant immune cells without instance segmentation. The workflow was developed as an ImageJ Macro available at https://github.com/CellProfiling/HDCA-FetalLung-SpatialProteomics/ (10.5281/zenodo.11650173). Briefly, the raw image stack was loaded and the user was prompted to select a region of interest, to which all analysis including threshold determination would be restricted. Next, the channel images for ACTA2, CD144, and CD45 were extracted, pre-processed, and subjected to semantic segmentation using ImageJ's Triangle threshold algorithm (for more details see Methods). Masks derived from ACTA2 and CD144 channel images were combined using the logical AND operation, and the resulting mask was filtered with a size filter: all objects smaller than 2000 px were removed. After this step, the mask was displayed to the user asking to validate it and to eventually add any missed-out artery regions or to remove artifacts. The objects in the validated mask were expanded within the ACTA2 mask to extend to the whole

artery region. The user was asked again for validation. Then, the expanded validated mask was expanded again using a maximum filter (radius of 80 px = 40 μm) to cover the peri-arterial regions. This mask was then used to dissect the immune cell mask coming from the CD45 channel into artery-close and artery-distant regions. The artery-close and the -distant mask were used to determine the mean and median intensity within -close and -distant immune cell regions, in all image channels of the raw 30-plex image. The example lung tissue image shown in the workflow is from the 11-pcw-old sample.

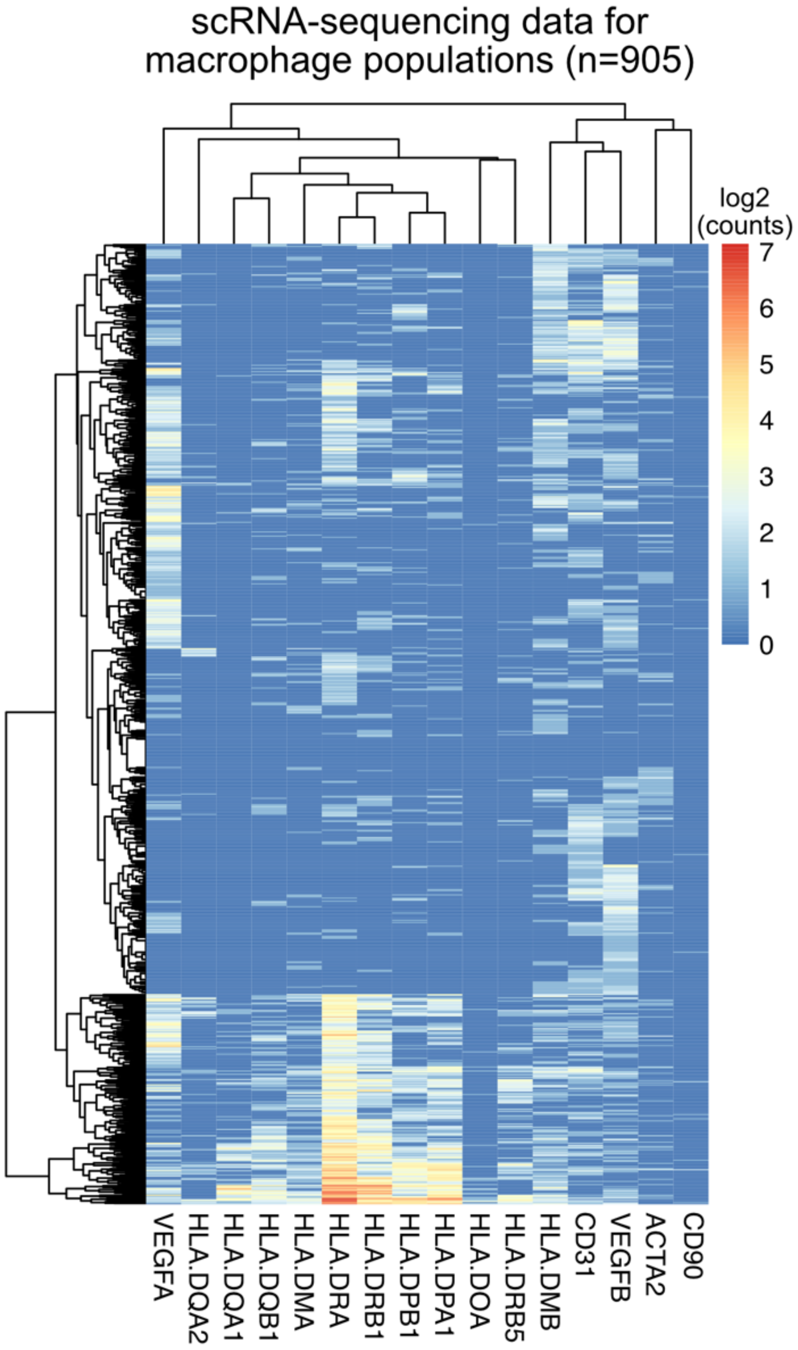

Supplementary Figure 24. Heatmap summarizing the expression patterns of selected genes in macrophage populations which were identified in single-cell transcriptomics human developing lung dataset by Sountoulidis *et al*[1]. A total of 905 cells (on the y-axis) were annotated as macrophages across the developmental weeks from 5 to 14. The heatmap summarizes the log2(normalized UMI counts+1) for 16 selected genes.

REFERENCES:

1 Sountoulidis, A. *et al.* A topographic atlas defines developmental origins of cell heterogeneity in the human embryonic lung. *Nature Cell Biology* **25**, 351-365 (2023). https://doi.org/10.1038/s41556-022-01064-x

2 Tyanova, S. *et al.* The Perseus computational platform for comprehensive analysis of (prote)omics data. *Nature Methods* **13**, 731-740 (2016). https://doi.org/10.1038/nmeth.3901

3 Zhang, Y., Parmigiani, G. & Johnson, W. E. ComBat-seq: batch effect adjustment for RNA-seq count data. *NAR Genomics and Bioinformatics* **2**, lqaa078 (2020). https://doi.org/10.1093/nargab/lqaa078
