# Supplementary material for: High-parametric protein maps reveal the spatial organization in early-developing human lung
Source: Nat Commun. 2024 Oct 30;15:9381. doi: 10.1038/s41467-024-53752-x (PMC11525936; doi:10.1038/s41467-024-53752-x)
Supplement: Supplementary file 3 — Reporting Summary [file 41467_2024_53752_MOESM3_ESM.pdf]

nature portfolio

Corresponding author(s): Burcu Ayoglu & Emma Lundberg

Last updated by author(s): Aug 27, 2024

Reporting Summary

Nature Portfolio wishes to improve the reproducibility of the work that we publish. This form provides structure for consistency and transparency in reporting. For further information on Nature Portfolio policies, see our Editorial Policies and the Editorial Policy Checklist.

Statistics

For all statistical analyses, confirm that the following items are present in the figure legend, table legend, main text, or Methods section.

n/a	Confirmed	
☐	☒	The exact sample size (*n*) for each experimental group/condition, given as a discrete number and unit of measurement
☐	☒	A statement on whether measurements were taken from distinct samples or whether the same sample was measured repeatedly
☐	☒	The statistical test(s) used AND whether they are one- or two-sided<br>*Only common tests should be described solely by name; describe more complex techniques in the Methods section.*
☒	☐	A description of all covariates tested
☐	☒	A description of any assumptions or corrections, such as tests of normality and adjustment for multiple comparisons
☐	☒	A full description of the statistical parameters including central tendency (e.g. means) or other basic estimates (e.g. regression coefficient) AND variation (e.g. standard deviation) or associated estimates of uncertainty (e.g. confidence intervals)
☐	☒	For null hypothesis testing, the test statistic (e.g. *F*, *t*, *r*) with confidence intervals, effect sizes, degrees of freedom and *P* value noted<br>*Give P values as exact values whenever suitable.*
☒	☐	For Bayesian analysis, information on the choice of priors and Markov chain Monte Carlo settings
☒	☐	For hierarchical and complex designs, identification of the appropriate level for tests and full reporting of outcomes
☐	☒	Estimates of effect sizes (e.g. Cohen's *d*, Pearson's *r*), indicating how they were calculated

Our web collection on statistics for biologists contains articles on many of the points above.

Software and code

Policy information about availability of computer code

Data collection	Fully automated imaging was performed using the PhenoCycler Fusion system (Akoya Biosciences). Raw 16-bit images were generated for each cycle using 20x magnification (resolution: 0.51um/pixel). Acquired images were processed by the PhenoImager Fusion software (Akoya Biosciences, v 1.0.3) and 8-bit ".qptiff"-image files were obtained as output for subsequent analyses.
Data analysis	Segmentation of fluorescence images was performed using PIPEX: https://github.com/CellProfiling/pipex, archieved in Zenodo (10.5281/zenodo.11642375)<br><br>For analysis of segmented imaging data, following Python packages have been used: anndata 0.8.0, scanpy 1.9.1, squidpy 1.2.2, numba 0.54.1, pandas 1.2.4, seaborn 0.11.2, matplotlib 3.5.0, numpy 1.20.3, scipy 1.7.3, pytometry 0.1.3, banksy 1.1.1.<br><br>Code Availability: The code for all programmatic analysis performed in this study is available in the following GitHub repository: https://github.com/CellProfiling/HDCA-FetalLung-SpatialProteomics/, archived in Zenodo (10.5281/zenodo.11650173)<br><br>The browser-based representation of the data was made available with the TissUUmaps tool: https://tissuumaps.github.io/

For manuscripts utilizing custom algorithms or software that are central to the research but not yet described in published literature, software must be made available to editors and reviewers. We strongly encourage code deposition in a community repository (e.g. GitHub). See the Nature Portfolio guidelines for submitting code & software for further information.

Data

Policy information about availability of data

All manuscripts must include a data availability statement. This statement should provide the following information, where applicable:

- Accession codes, unique identifiers, or web links for publicly available datasets
- A description of any restrictions on data availability
- For clinical datasets or third party data, please ensure that the statement adheres to our policy

Processed fluorescent imaging datasets can be accessed at Zenodo in .qptiff files format together with selected set of metadata for each individual week (10.5281/zenodo.11652584).

Raw-image datasets (807GB) are available from the corresponding authors on request because of data size limitations.

Segmentation output in .csv file format used for all the presented downstream analyses are available at Zenodo (10.5281/zenodo.11623168).

Cell type annotations resulting from downstream analyses and marker intensity plots, both overlaid on DAPI channel images for each week, can be accessed under the "Spatial Proteomics" tab at the following interactive portal: https://hdca-sweden.scilifelab.se/tissues-overview/lung/.

Research involving human participants, their data, or biological material

Policy information about studies with human participants or human data. See also policy information about sex, gender (identity/presentation), and sexual orientation and race, ethnicity and racism.

Reporting on sex and gender	At time of the collection of embryonic /fetal tissue donors, the sex of the embryos/fetuses was not determined, thus no biased selection was performed. Subsequent transcriptomics analyses on other organs from these embryos revealed the sex of three out of five embryos/fetuses that provided the analyzed and described lung tissue in this study: The 8.5 and 13 post conception week (pcw) donors were female and the 11 pcw donor was male. Information regarding the sex of the 6 pcw and 12 pcw is currently not available as the organs from these specific donors have not been sequenced yet. The findings of the study are not restricted to only one sex.
Reporting on race, ethnicity, or other socially relevant groupings	No socially constructed or socially relevant categorization of variables was used in this study.
Population characteristics	At time of the collection of embryonic /fetal tissue donors, the sex of these embryos/fetuses were not determined. Subsequent transcriptomics studies on the other organs of these embryos determined the sex of the three out of five embryos/fetuses providing the analyzed and described lung tissue in this study: 8.5 and 13 post conception week (pcw) donors were females and 11 pcw donor was male. Information regarding the sex of the 6 pcw and 12 pcw is not available as the organs from these specific donors have not been sequenced. Findings of the study do not apply to only one sex.
Recruitment	The tissue donors were recruited among pregnant women who had decided to terminate their pregnancy. Referral to hospitals was managed by a central office for all abortion clinics in the Stockholm region, and according to our information, this process was random. Recruitments were conducted by midwives who were not involved in the research, ensuring no bias in the selection of participants. No compensation of any kind was provided to the tissue donors.<br>Inclusion criteria were: 18 years of age or older, fluency in Swedish. Exclusion criteria were: Abortions performed for medical reasons, by socially compromised women and/or women demonstrating any signs that the consent may not be informed.
Ethics oversight	The use of human embryonic/fetal material from the elective abortions was approved by the Swedish National Bord of Health and Welfare (Socialstyrelsen), and the molecular analysis of this material was approved by the Swedish Ethical Review Authority (2018/769-31). The clinical staff acquired informed written consent from the tissue donors, that the retrieved embryonic/fetal material can be used for research purposes and that they are able to withdraw their consent anytime.

Note that full information on the approval of the study protocol must also be provided in the manuscript.

Field-specific reporting

Please select the one below that is the best fit for your research. If you are not sure, read the appropriate sections before making your selection.

☒ Life sciences ☐ Behavioural & social sciences ☐ Ecological, evolutionary & environmental sciences

For a reference copy of the document with all sections, see nature.com/documents/nr-reporting-summary-flat.pdf

Life sciences study design

All studies must disclose on these points even when the disclosure is negative.

Sample size	No sample-size calculation was performed to statistically predetermine the sample size. The samples used in these study were rarely available human embryonic/fetal lungs extracted from human embryonic/fetal donors. Series of intact embryonic lung whole tissue sections from all five human embryonic donors, each representing a distinct developmental timepoint, were used for the study. Given the difficulty of

procuring such samples, we performed the study with this limited cohort size.

Data exclusions

No tissue samples were excluded from the analysis. Certain tissue sample image regions, belonging either to non-lung tissue, or demonstrating staining or imaging artifacts, were excluded from the downstream quantitative analysis. This is shown in detail in Supplementary Figure 4 and also discussed in Supplementary Information file. The criterion for exclusion of non-lung regions was pre-established. The criterion for exclusion of staining and/or imaging artifact regions was established after image acquisition.

Replication

For each antibody in the 30-plex panel, staining was replicated at least three times: minimum twice during antibody validation with regular IF tests and minimum once in the final multiplexed assay. Dozens of consecutive sections from each tissue sample were used to screen over 50 antibodies, ultimately forming the final 30-plex panel. The inclusion or exclusion of antibodies in the presented final 30-panel was based on both antibody performance and the consistent replication of staining patterns compared to regular IF. Additionally, the protein-level results from antibodies were corroborated against spatially resolved transcriptomics and single-cell RNA sequencing datasets from the partially overlapping sample cohort by Sountoulidis et al., 2023.

Randomization

Randomization was not relevant for this study as the study was based on the analysis of five different human embryonic samples at five different post conception weeks.

Blinding

Blinding was not relevant for this study as the study was based on the analysis of five different human embryonic lung samples at five different post conception weeks.

Reporting for specific materials, systems and methods

We require information from authors about some types of materials, experimental systems and methods used in many studies. Here, indicate whether each material, system or method listed is relevant to your study. If you are not sure if a list item applies to your research, read the appropriate section before selecting a response.

Materials & experimental systems

n/a	Involved in the study
☐	☒ Antibodies
☒	☐ Eukaryotic cell lines
☒	☐ Palaeontology and archaeology
☒	☐ Animals and other organisms
☒	☐ Clinical data
☒	☐ Dual use research of concern
☒	☐ Plants

Methods

n/a	Involved in the study
☒	☐ ChIP-seq
☒	☐ Flow cytometry
☒	☐ MRI-based neuroimaging

Antibodies

Antibodies used

Antibodies oligo-conjugated in-house:

*Anti EPCAM mouse monoclonal antibody (Thermo Fisher ,1B7, #14932682, working dilution: 1:400)- conjugated with BX042 (Akoya Biosciences, 5550015): https://www.thermofisher.com/antibody/product/CD326-EpCAM-Antibody-clone-1B7-Monoclonal/14-9326-82
*Anti CD123 mouse monoclonal antibody (Thermo Fisher, 6H6, # 14-1239-82, wd: 1:100)- conjugated with BX054 (Akoya Biosciences, 5550019): https://www.thermofisher.com/antibody/product/CD123-Antibody-clone-6H6-Monoclonal/14-1239-82
*Anti SOX2 rat monoclonal antibody (Thermo Fisher ,Btjce, 14-9811-82, working dilution: 1:200)- conjugated with BX024 (Akoya Biosciences, 5550010): https://www.thermofisher.com/antibody/product/SOX2-Antibody-clone-Btjce-Monoclonal/14-9811-82
*Anti CD144 mouse monoclonal antibody (Thermo Fisher, 16B1, # 14-1449-82, wd: 1:100)- conjugated with BX016 (Akoya Biosciences, 5150001): https://www.thermofisher.com/antibody/product/CD144-VE-cadherin-Antibody-clone-16B1-Monoclonal/14-1449-82
*Anti ACTA2 mouse monoclonal antibody (Abcam, ab119952,4A4, working dilution: 1:70)- conjugated with BX028 barcode (5150005): https://www.abcam.com/en-us/products/primary-antibodies/pan-actin-antibody-4a4-ab119952
*Anti CD68 mouse monoclonal (Thermo Fisher, KP1, # 14-0688-82, wd: 1:100)- conjugated with BX010(Akoya Biosciences, 5450016): https://www.thermofisher.com/antibody/product/CD68-Antibody-clone-KP1-Monoclonal/14-0688-82
*Anti CD44 rat monoclonal antibody(Thermo Fisher, Hermes-1, # MA4400, wd: 1:100): https://www.thermofisher.com/antibody/product/CD44-Antibody-clone-Hermes-1-Monoclonal/MA4400
*Anti DCN rabbit polyclonal antibody (Atlas Antibodies , HPA003315, working dilution: 1:50)- conjugated with BX013 (Akoya Biosciences, 5450017): https://www.atlasantibodies.com/products/primary-antibodies/triple-a-polyclonals/anti-dcn-antibody-hpa003315-100ul/?language=en
*Anti WT1 mouse monoclonal antibody (Thermo Fisher, 6F-H2, # MA1-46028, working dilution: 1:50)- conjugated with BX006 (Akoya Biosciences, 5550018): https://www.thermofisher.com/antibody/product/WT1-Antibody-clone-6F-H2-Monoclonal/MA1-46028
*Anti CD163 mouse monoclonal antibody (Thermo Fisher, GHI/16, # MA5-17716, wd: 1:50)- conjugated with BX005 (Akoya Biosciences, 5450024): https://www.thermofisher.com/antibody/product/CD163-Antibody-clone-GHI-61-Monoclonal/MA5-17716
*Anti COL1A1 mouse monoclonal antibody (R&D systems ,#816161, MAB6220-100, working dilution: 1:300)- conjugated with BX002 (Akoya Biosciences, 5450023): https://www.rndsystems.com/products/human-collagen-i-alpha1-antibody-816154_mab62202?gad_source=1&gclid=CjwKCAjwuMC2BhA7EiwAmJKRrIBjYw7softxEunE9Oxk_PEllm7D3sA6tq4rsC6FGuLgvqDIOKC37BoC9XMQAvD_BwE&gclsrc=aw.ds

*Anti CD56 rabbit monoclonal antibody (Abcam, CAL53, ab251595, working dilution: 1:50)- conjugated with BX029 (Akoya Biosciences, 5250005): https://www.abcam.com/en-us/products/primary-antibodies/ncam1-antibody-cal53-bsa-and-azide-free-ab251595
*Anti MRC1 rabbit polyclonal antibody (Abcam, ab64693, working dilution: 1:100)- conjugated with BX030 (Akoya Biosciences, 5550012): https://www.abcam.com/en-us/products/primary-antibodies/mannose-receptor-antibody-ab64693
*Anti Vimentin mouse monoclonal antibody (BioLegend,O91D3, 677802, working dilution: 1:300)- conjugated with BX045 (Akoya Biosciences, 5550016): https://www.biolegend.com/en-gb/products/purified-anti-vimentin-antibody-12022
*Anti PRX rabbit monoclonal antibody (Abcam ,EPR24150-36, ab27808, working dilution: 1:100)- conjugated with BX052 (Akoya Biosciences,5250012): https://www.abcam.com/en-us/products/primary-antibodies/prx-antibody-epr24150-36-bsa-and-azide-free-ab278083
*Anti CLDN5 mouse monoclonal antibody (Thermo Fisher ,4C3C2, # 35-2500, working dilution: 1:100)- conjugated with BX041 (Akoya Biosciences, 5250008): https://www.thermofisher.com/antibody/product/Claudin-5-Antibody-clone-4C3C2-Monoclonal/35-2500
*Anti SOX9 goat polyclonal antibody (R&D systems , AF3075, working dilution: 1:250)- conjugated with BX033 (Akoya Biosciences, 5550013): https://www.rndsystems.com/products/human-sox9-antibody_af3075?gad_source=1&gclid=CjwKCAjwuMC2BhA7EiwAmJKRrOousEotXEa3_iyGNiP0qkhlp3YQVc5HwSrr9gWHckL5DCD2cVQvahoCOSYQAvD_BwE&gclsrc=aw.ds
*Anti TTF1 mouse monoclonal antibody (Abcam ,EP1584Y, ab216648, working dilution: 1:100)- conjugated with BX007 (Akoya Biosciences, 5450015): https://www.abcam.com/en-us/products/primary-antibodies/ttf1-nkx2-1-antibody-ep1584y-bsa-and-azide-free-ab216648

Antibodies used as pre-conjugated (catalog numbers at the time of use were later updated by the manufacturer and can be found in the provided links):

*Anti CD3 antibody (Akoya Biosciences, 4350008, wd: 1:200): https://my.akoyabio.com/ccrz__ProductDetails?sku=4550103&cclcl=en_US
*Anti CD4 antibody(Akoya Biosciences, 4350010, wd: 1:200): https://my.akoyabio.com/ccrz__ProductDetails?sku=4550105
*Anti CD19 antibody (Akoya Biosciences,4350003, wd: 1:100): https://my.akoyabio.com/ccrz__ProductDetails?sku=4550099&cclcl=en_US
*Anti CD31 antibody (Akoya Biosciences, 4250009, wd:1:200): https://my.akoyabio.com/ccrz__ProductDetails?sku=4250009&cclcl=en_US
*Anti CD34 antibody (Akoya Biosciences, 4250020, wd: 1:200): https://my.akoyabio.com/ccrz__ProductDetails?sku=4250020&cclcl=en_US
*Anti CD45 antibody (Akoya Biosciences,4450003, wd: 1:100): https://my.akoyabio.com/ccrz__ProductDetails?sku=4450003&cclcl=en_US
*Anti CD90 antibody (Akoya Biosciences,4150021, wd: 1:200): https://my.akoyabio.com/ccrz__ProductDetails?sku=4150021&cclcl=en_US
*Anti PDPN antibody (Akoya Biosciences, 4250004, wd: 1:200): https://my.akoyabio.com/ccrz__ProductDetails?sku=4250004&cclcl=en_US
*Anti HLA-DR antibody (Akoya Biosciences, 4250006, wd: 1:200): https://my.akoyabio.com/ccrz__ProductDetails?sku=4250006&cclcl=en_US
*Anti Ki67 antibody (Akoya Biosciences, 4250019, wd: 1:200): https://my.akoyabio.com/ccrz__ProductDetails?sku=4250019&cclcl=en_US
*Anti Pan-cytokeratin antibody (Akoya Biosciences, 4150020, wd: 1:300): https://my.akoyabio.com/ccrz__ProductDetails?sku=4150020&cclcl=en_US
*Anti E-cadherin antibody (Akoya Biosciences, 4250021, wd: 1:200): https://my.akoyabio.com/ccrz__ProductDetails?sku=4250021

Supplementary Table 1 lists an additional set of antibodies and their catalog numbers that were screened beyond these 30 antibodies, although their data was not reported and discussed in the manuscript.

Validation

For all the antibodies, manufacturers had provided information regarding the species-reactivity and citation(s) for usage on tissue sections for immunofluorescence. Supplementary Table 1 of the manuscript provides further information such as tested tissue type and tested dilution(s) regarding the in-house validation efforts. Figure 1F reports the staining patterns for panel markers in a selected tissue region of a 8.5-week-old lung sample.

Plants

Seed stocks

Report on the source of all seed stocks or other plant material used. If applicable, state the seed stock centre and catalogue number. If plant specimens were collected from the field, describe the collection location, date and sampling procedures.

Novel plant genotypes

Describe the methods by which all novel plant genotypes were produced. This includes those generated by transgenic approaches, gene editing, chemical/radiation-based mutagenesis and hybridization. For transgenic lines, describe the transformation method, the number of independent lines analyzed and the generation upon which experiments were performed. For gene-edited lines, describe the editor used, the endogenous sequence targeted for editing, the targeting guide RNA sequence (if applicable) and how the editor was applied.

Authentication

Describe any authentication procedures for each seed stock used or novel genotype generated. Describe any experiments used to assess the effect of a mutation and, where applicable, how potential secondary effects (e.g. second site T-DNA insertions, mosiacism, off-target gene editing) were examined.
